# Intragrain impurity annihilation for highly efficient and stable perovskite solar cells

**DOI:** 10.1038/s41467-024-46588-y

**Published:** 2024-03-14

**Authors:** Songhua Cai, Zhipeng Li, Yalan Zhang, Tanghao Liu, Peng Wang, Ming-Gang Ju, Shuping Pang, Shu Ping Lau, Xiao Cheng Zeng, Yuanyuan Zhou

**Affiliations:** 1 Department of Applied Physics, The Hong Kong Polytechnic University, Kowloon, Hong Kong SAR, China; 2 Qingdao Institute of Bioenergy & Bioprocess Technology, Chinese Academy of Sciences, Qingdao, Shandong 266101 China; 3 Department of Chemical and Biological Engineering, The Hong Kong University of Science and Technology, Clear Water Bay, Hong Kong SAR, China; 4 Department of Physics, Hong Kong Baptist University, Kowloon, Hong Kong SAR, China; 5 Department of Physics, University of Warwick, Coventry, CV4 7AL UK; 6 Key Laboratory of Quantum Materials and Devices of Ministry of Education, School of Physics, Southeast University, Nanjing, 211189 China; 7 Department of Materials Science and Engineering, City University of Hong Kong, Kowloon, Hong Kong SAR, China

**Keywords:** Solar cells, Organic-inorganic nanostructures, Devices for energy harvesting

## Abstract

Intragrain impurities can impart detrimental effects on the efficiency and stability of perovskite solar cells, but they are indiscernible to conventional characterizations and thus remain unexplored. Using in situ scanning transmission electron microscopy, we reveal that intragrain impurity nano-clusters inherited from either the solution synthesis or post-synthesis storage can revert to perovskites upon irradiation stimuli, leading to the counterintuitive amendment of crystalline grains. In conjunction with computational modelling, we atomically resolve crystallographic transformation modes for the annihilation of intragrain impurity nano-clusters and probe their impacts on optoelectronic properties. Such critical fundamental findings are translated for the device advancement. Adopting a scanning laser stimulus proven to heal intragrain impurity nano-clusters, we simultaneously boost the efficiency and stability of formamidinium-cesium perovskite solar cells, by virtual of improved optoelectronic properties and relaxed intra-crystal strain, respectively. This device engineering, inspired and guided by atomic-scale in situ microscopic imaging, presents a new prototype for solar cell advancement.

## Abstract (web summary)

The detrimental effects of intragrain impurity nanoclusters on the efficiency and stability of perovskite solar cells remain unexplored. Here, the authors study the intragrain impurity annihilation by in situ scanning transmission electron microscopy and adopt a laser stimulus to heal such impurity.

## Introduction

Perovskite solar cells (PSCs) have attracted enormous attention in the field of photovoltaic energy due to their high power conversion efficiencies (PCEs) which have rapidly reached certified 26.2%^1,2^. The technological advance of PSCs has been largely driven by the development of various microstructural tailoring strategies that can reduce defects and impurities generated during the processing, post-synthesis storage, and device operation^3–10^. Because metal halide perovskite thin films in state-of-the-art PSCs are polycrystalline, and they are generally grown via solution crystallization routes, defects, and impurities are intuitively considered to exist primarily in the inter-grain regions^11–14^. Therefore, in the past years, research effort has primarily been devoted to tailoring grain boundaries (GBs) for favorable defect properties and phase distribution. Nevertheless, the fundamental behaviors and effects of intragrain defects and impurities have been rarely studied, although they have been known essential to semiconductors in general^15,16^. One major hurdle in this research direction is to reliably image the microstructure and dynamics of intragrain defects and impurities in perovskites^3,17–20^. Transmission electron microscopy (TEM) is a workhorse tool for materials characterization with high spatial resolution, which has been leveraged to demonstrate unambiguous observation of atomic-scale details of perovskites^21–25^. In a recent study, we reported a simple, modified scanning TEM (STEM) method in conjunction with focus ion beam (FIB) nanofabrication of perovskite sample specimen, which allows direct, reliable observation of perovskite microstructures that are embedded in PSCs^26^. By leveraging this significant development, herein we demonstrate an in situ STEM approach to visualize the atomic-scale microstructural evolution in PSCs with a controllable, low-dose electron beam as the external energy stimulus. We first revealed that nanoscale impurity phases, primarily in the form of PbI_2_, can frequently exist in the intragrain regions (within the crystalline perovskite matrix) of the solution-processed formamidinium-cesium (FA-Cs) PSCs. Counterintuitively, upon the external energy stimulus (i.e., controlled STEM electron beam), these intragrain impurity phases are readily healed to perovskites via versatile crystallographic transformation modes which are atomically resolved. The dynamic structural and electronic landscapes of such intragrain impurity annihilation (IGIA) are further elaborated using theoretical calculations based on equivalent, correlated models.

The IGIA revealed by STEM has inspired the adoption of an optimal laser stimulus to treat as-fabricated FA-Cs perovskite films, which boosts the PCEs of resulting PSCs. We further confirmed that the laser-induced IGIA underpins the device enhancement regardless of the difference in the stimulus type (laser *versus* electron beam). The phase-healed perovskite grains also exhibit relaxed intra-crystal strain structures, thus contributing to higher device stability in PSCs. Furthermore, it is feasible to adopt the laser-induced IGIA to recover the performance of PSCs even during device storage. We combined theoretical and experimental methods to gain insights into the connection between our in situ observation and device engineering, pointing to a promising methodology featuring the translation of atomic-level fundamental studies to module-level device technologies.

## Results

**Fig. 1:**
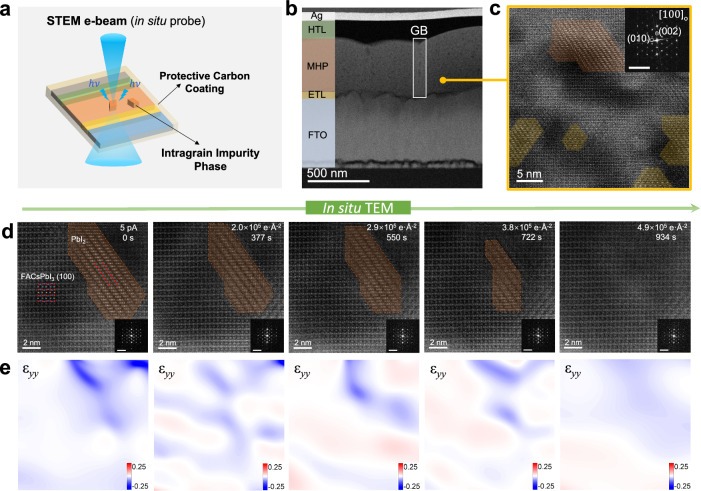
In situ STEM observation of intragrain impurity annihilation in the PSC. **a** Experimental flow for in situ STEM observation: The STEM electron beam was used for structural characterization and also act as an in situ stimulus, finally, the crystallographic transition of different phases can be stimulated by high-energy electron probe. **b** STEM image of the starting PSC cross-sectional specimen, shows the perovskite layer consisting monolayer perovskite grains and grain boundaries. **c** High-resolution STEM-HAADF image of the initial PSC sample. Orange and yellow regions represent impurity phases of different types that exist in the film. The inset is the corresponding FFT pattern, indicating the [100]_o_ projection direction, scale bar: 5 nm^−^^1^. **d** In situ observation of transformation from PbI_2_ nanocluster to perovskite phase. Atomic resolution STEM-HAADF images of orthorhombic (FA,Cs)PbI_3_ nano-region projected along [100]_o_ direction contains a single PbI_2_ nanocluster (marked by orange dashed lines). The transformation process of the PbI_2_ nanocluster was triggered by continuous electron probe scanning. Insets are corresponding FFT patterns of each STEM-HAADF image, scale bar: 4 nm^−^^1^. **e** Corresponding out-of-plane strain ε_*yy*_ distributions of d generated by a GPA analysis, the intragrain strain generated from PbI_2_ nanocluster can be effectively relaxed by IGIA.

The experimental flow for performing the in situ STEM observation is schematically illustrated in Fig. 1a and Supplementary Fig. 1. Briefly, we fabricated an FA-Cs PSC with the optimal synthesis conditions (details in Methods) and prepared the device cross-section lamella using FIB nanofabrication, immediately followed by the sputtering deposition of a 10 nm thick amorphous carbon layer onto both sides of the lamella (Supplementary Fig. 1). Then, as illustrated in Fig. 1a, we used a controlled electron dose to trigger the evolution of intragrain microstructure in the PSC cross-section and acquired a sequence of STEM images at different beam irradiations to resolve the perovskite intragrain crystallographic transformation. Note that high-energy electron beam is a widely used in situ stimulus for specimen morphology modifications and triggering structure evolutions inside TEM^27–29^.

Figure 1b is a low-magnification STEM-HAADF image acquired with a beam current of the electron probe of 10 pA, exhibiting the initial microstructure of the PSC sample. Using energy dispersive spectroscopy (EDS), each layer of this PSC device can be delineated, confirming the sample integrity (Supplementary Fig. 2a). We then reduced the beam current of the electron probe to 5 pA to acquire high-resolution STEM-HAADF images. The grain interior shows a clear lattice structure. Based on the real-space image and corresponding fast Fourier transform (FFT) pattern (Fig. 1c), this perovskite grain is resolved as an orthorhombic phase (space group *Pnma*) projected along [100]_o_ direction (Supplementary Fig. 2b)^30^. A close examination of the high-resolution STEM-HAADF image shown in Fig. 1c reveals at least two types of nanoscale intragrain impurity phases, PbI_2_ (orange regions) and non-PbI_2_ impurity (yellow region; later resolved as an immediate phase with 0D crystallographic structure), have been populated and distributed within the perovskite grain. The formation of these intragrain impurities may be influenced by several factors, especially for the fabrication and storage conditions. For the FA-Cs perovskite specimen prepared with a much longer annealing time than the optimal condition, a higher density of intragrain impurities can be observed (Supplementary Fig. 3b). Similarly, the under-annealed FA-Cs perovskite specimen also exhibits an abundance of intragrain impurity nanoclusters (Supplementary Fig. 4). In addition, the storage of vacuum packed FIBed FA-Cs perovskite device cross-section specimens can also induce the formation of intragrain impurities (Supplementary Fig. 5). As a result, both the poorly-prepared perovskite films and degraded perovskite specimens will incorporate a considerable density of intragrain impurities, although these intragrain impurity nanoclusters may originate from different sources. Supplementary Figs. 6 and 7 show more atomic-level details of such intragrain impurities. Nevertheless, this nanoscale phase variation can hardly be identified only from the FFT pattern (Fig. 1d inset) without real-space images, because of the similarity in spatial symmetry of their structures. It is worth noting that the intragrain PbI_2_ impurity nanoclusters can also exist in the methylammonium-cesium (MA-Cs) perovskite (Supplementary Fig. 8c, d), similar to the case of FA-Cs perovskite (Supplementary Fig. 8a, b), which implies the generality of this intragrain phenomena.

Herein we note that with the protection of amorphous carbon layers, no obvious changes occur in cross-sectional FA-Cs PSC specimens during STEM observation and imaging which only needs a short scanning time of electron probe. This unlocks investigations of dynamic phenomena induced by continuous electron beam irradiation. In this context, an electron probe with a similar 5 pA beam current was used for in situ scanning of FA-Cs PSC cross-section specimens. Figure 1d shows an orthorhombic perovskite grain projected along [100]_o_ direction, a nano-region with PbI_2_ (space group *R3mH*, projected along [110]_o_ direction^31^, marked by orange as region 2) was selected. With the following continuous scanning of the focused electron probe (average dose rate calculated as 525 e·Å^−^^2^/s), some remarkable structural evolutions were recorded at the atomic level (Fig. 1d), revealing the unprecedented transformation details from PbI_2_ to perovskite phase in the IGIA process. Supplementary Fig. 9 demonstrates the shrinkage trend of the PbI_2_ region with respect to the accumulated electron dose. At first, interestingly, little change occurs on this PbI_2_ nanocluster after the first 248 s electron probe scanning (about 1.3 × 10^5^ e·Å^−^^2^ accumulated dose). This phenomenon further implies the lowest reaction rate with respect to the smallest specific surface area of the PbI_2_ nanocluster at the preliminary stage. Subsequently, the shrinkage of the PbI_2_ nanocluster accelerates in both directions after the accumulated electron dose exceeds 1.3 × 10^5^ e·Å^−^^2^ and is finally completely transferred to the perovskite phase. The FFT patterns (Fig. 1d insets) also show no noticeable changes in this process. Although the quantified volume of the PbI_2_ region can hardly be obtained only from this projection-view STEM image, a qualitative analysis is possible as the specific surface area is inversely proportional to the volume of the cluster. Therefore, this IGIA process in FA-Cs perovskite should have a reaction rate proportional to the specific surface area. Generally speaking, the initial large cluster with a small specific surface area will exhibit a slower reaction rate. As a result, with the cluster shrinking in this process, the increasing specific surface area will facilitate the reaction propagation. In addition, we found the existence of the PbI_2_ nanocluster leads to the remarkable intragrain strain, which may harm the perovskite performance (Fig. 1e). This intragrain strain can be effectively relaxed by the IGIA process (Fig. 1e).

In another case, a nano-region with both PbI_2_ and non-PbI_2_ impurity phase was selected as shown in Supplementary Fig. 10. This non-PbI_2_ impurity phase can be resolved as a possible zero-dimensional cation-intercalated PbI_6_ octahedral with a chemical composition of (FA,Cs)_4_PbI_6_ (Supplementary Fig. 7)^32–34^. Although (FA,Cs)_4_PbI_6_ has not been reported previously as a bulk material, the nano-confinement of this phase within the crystalline perovskite matrix may enable its meta-stability. In this case, the non-PbI_2_ impurity phase nanocluster at the top right corner shrinks (region 1) and is then completely transferred to the perovskite phase right after being irradiated by an electron probe (Supplementary Fig. 10d, i). During this period, the middle PbI_2_ nanocluster (region 2) exhibits little evident change, with the left side non-PbI_2_ impurity phase nanocluster (region 3) shrinking slightly. After scanning for 479 s, the shrinkage of both regions 2 and 3 begins to accelerate. The prior transformation of region 1 (non-PbI_2_ impurity phase) demonstrates the intermediateness of this phase than PbI_2_. Besides, the shrinkage rate also tends to accelerate with the keeping decrease of PbI_2_ and non-PbI_2_ impurity phase areas (Supplementary Fig. 10i). Note if only from the FFT patterns (insets in Supplementary Fig. 10), no obvious changes can be identified. The lower shrinkage pace of region 3 than region 1 may be attributed to the much larger size and smaller specific surface area. Besides, we also find the existence of a non-PbI_2_ impurity phase will lead to additional intragrain strain (Supplementary Fig. 11b). Similarly, this intragrain strain can also be effectively relaxed by the IGIA process (Supplementary Fig. 11d), which may contribute to improved stabilization in the resultant device^35–37^.

**Fig. 2:**
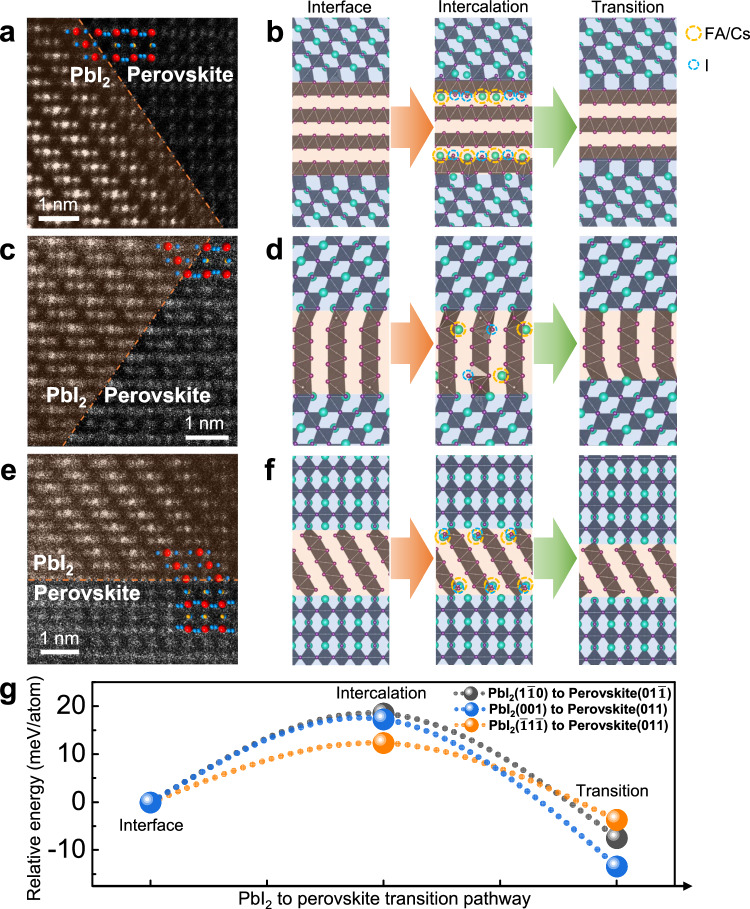
Mechanistic insights into the intragrain impurity annihilation in PSCs. **a**, **c**, **e** Atomic resolution STEM-HAADF images of intragrain PbI_2_ impurity (marked by orange region) and perovskite interfaces along perovskite (01$\bar{1}$), (011) and (001) plane, respectively. **b**, **d**, **f** Modeled progression of phase transformation from PbI_2_ sheet structures with octahedra edge-sharing connectivity to the perovskite structures with octahedra corner-sharing connectivity by intercalation of FA/Cs and I originated from external energy stimulus in different PbI_2_-perovskite interfaces in accordance with (**a**, **c**, **e**) respectively. **g** Calculated relative energy difference between the initial interface with higher proportions of PbI_2_ sheet, intermediate structure with intercalation of FA/Cs and I, and final transition from PbI_2_ sheet to perovskite structures for IGIA phase transformation at different interfaces. The intercalations of FA/Cs and I are highlighted by the orange and blue dashed circle.

Our in situ observations reveal clear an IGIA of intragrain PbI_2_ nanocluster to perovskite phase. As shown in Fig. 1d, the IGIA propagates with the continuous shrinkage of the PbI_2_ nanocluster. Interestingly, the IGIA reaction rate also exhibits an obvious specific surface area dependency. This implies an interface-dependent reaction mechanism, that a step-by-step interfacial transition from PbI_2_ to perovskite with the intercalation of FA/Cs cations and I anions driven by electron irradiation. To investigate the underlying reaction mechanisms, the atomic-scale structures of PbI_2_/perovskite interfaces are resolved by atomic resolution STEM (Fig. 2a–c). Each type of interface can be formed between different crystallographic facets of PbI_2_ and perovskite phases. This implies a possible divergent reaction barrier and procedure for different orientated interfaces, which is critical for proposing plausible mechanisms. In this regard, we proposed different crystallographic pathways for the IGIA in PSCs, as schematically shown in Fig. 2. To estimate divergent reaction barriers of different IGIA routes, we probed the thermodynamics of the transformation between the interfaces comprised different crystallographic facets of PbI_2_ and perovskite phases using density functional theory (DFT). With the detailed atomic information via the STEM imaging, we considered three interfaces originating from the different crystallographic facets between PbI_2_ and perovskite, PbI_2_ ($1\bar{1}0$)-perovskite (01$\bar{1}$), PbI_2_ (001)-perovskite (011) and PbI_2_ ($\bar{1}1\bar{1}$)-perovskite (001) as the interface transition routes (Fig. 2b, d, f). We start from the plausible hypothesis that monovalent cation FA/Cs and anion I gradually intercalate between the edge-sharing layers of Pb-I octahedra in PbI_2_ driven by electron irradiation, leading to a step-by-step interfacial transition from PbI_2_ to perovskite. We manually constructed such a structure of interfaces comprising different proportions of PbI_2_ and perovskite, in which the starting mixed PbI_2_/perovskite phase contains a higher proportion of PbI_2_ and the final mixed PbI_2_/perovskite phase contains a lower proportion of PbI_2_, demonstrating a step-by-step interfacial transition. We estimated the thermodynamic cost to form an intermediated phase, which is constructed by intercalating FA/Cs and I ions in between the layers of PbI_2_ around the interface (Fig. 2b, d, e). These values provided a theoretical assessment of the reaction barriers of different IGIA routes. We obtain a barrier height of 18.5 meV per atom for the PbI_2_ ($1\bar{1}0$) to perovskite (01$\bar{1}$) interfacial transition, 17.3 meV per atom for the PbI_2_ (001) to perovskite (011) interfacial transition, and 12.4 meV per atom for PbI_2_ ($\bar{1}1\bar{1}$) to perovskite (001) interfacial transition, indicating that PbI_2_ ($\bar{1}1\bar{1}$)-perovskite (001) interfacial transition was a more favor IGIA pathway with respect to other pathways. In particular, when considering the transformation process of (FA,Cs)PbI_3_ perovskites, the barrier heights per formula unit become more relevant, ranging from 60 to 90 meV. We note that our in situ observations also show the possible higher reaction rate around the PbI_2_ ($\bar{1}1\bar{1}$)-perovskite (001) interface.

As demonstrated before, the reaction rate in the IGIA process should be strongly related to the specific surface area of the impurity nanoclusters. Although the specific surface area is related to several factors, i.e., the geometrical shape of the nanocluster, the size plays a more important role. For a larger PbI_2_ nanocluster (Supplementary Fig. 12), it takes a longer time to completely transfer to the perovskite phase than the case shown in Fig. 1d. If the PbI_2_ nanocluster is further enlarged, this IGIA will be much harder and needs more input energy (Supplementary Figs. 13, 14), which makes the generation and expansion of PbI_2_ clusters tend to be irreversible in perovskites under operation conditions. Therefore, this IGIA mechanism will be more effective for the nanoscale intermediate non-PbI_2_ phase and PbI_2_ nanoclusters exist in perovskite grains with external energy input. This also provides a possible explanation for the slight improvement of as-prepared PSC devices when starting operations^38^, as the photon irradiation may also trigger the IGIA process to eliminate the existing nanoscale impurity clusters. However, this process may not be enough to prevent the generation of large-scale PbI_2_ nanoclusters during long-period operations of PSCs.

**Fig. 3:**
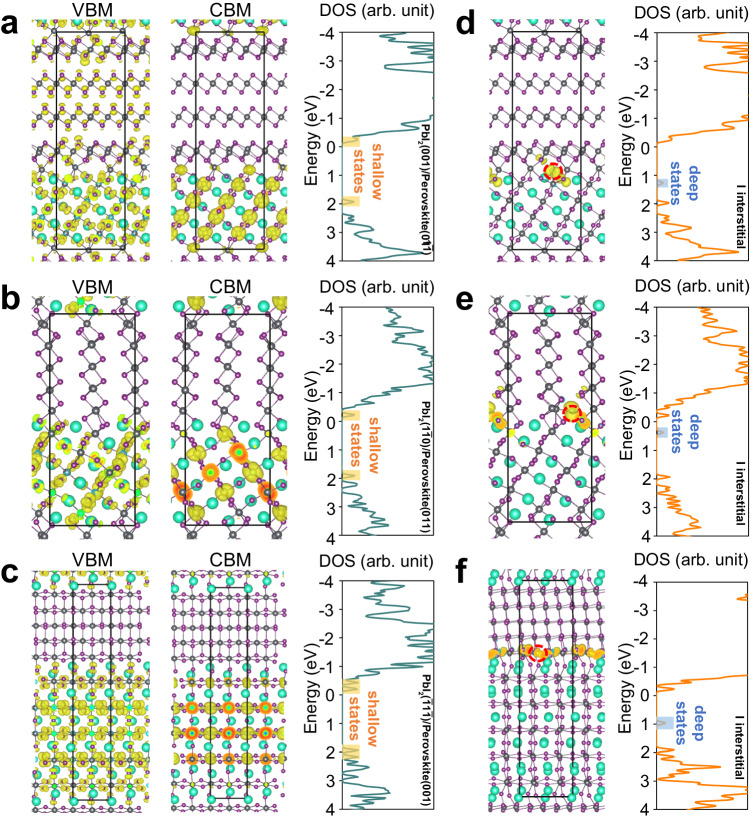
Calculated electronic and defect properties of the three interfaces formed by intragrain PbI_2_ impurity and perovskite phase. **a**–**c** The charge density corresponding to the VBM and CBM, the total DOS of the PbI_2_-perovskite interfaces along perovskite (01$\bar{1}$), (011), and (001) plane correlated to atomic resolution STEM-HAADF images, respectively. The cyan, black, and purple spheres denote FA/Cs, Pb, and I atoms, respectively, and the yellow areas denote the computed charge density. **d**–**f** Point defects of perovskites including vacancies, interstitials, and anti-sites. Considering the progression of the structural transition, I, Pb, Cs/FA interstitials, and I vacancies are focused in this study. The charge density (yellow area) of the shoulder peak near the band edges and total DOS for PbI_2_-perovskite interfaces corresponds to (**a**–**c**) with one I interstitials (per supercell), respectively. The I interstitials per supercell is highlighted by the red dashed circle.

To get insight into the impact of interfaces on the performance of devices, we explored the optoelectronic properties using first-principle calculations. Likewise, we considered three interfaces between PbI_2_ and perovskite, PbI_2_ ($1\bar{1}0$)-perovskite (01$\bar{1}$), PbI_2_ (001)-perovskite (011) and PbI_2_ ($\bar{1}1\bar{1}$)-perovskite (001) (see Fig. 3a–c). For the interface of the PbI_2_ ($1\bar{1}0$)-perovskite (01$\bar{1}$), the valence band maximum (VBM) is contributed from both the PbI_2_ and perovskite and the conduction band minimum (CBM) is mainly contributed from the perovskite component (Fig. 3a), with slightly more deep states in the band gap. Nevertheless, from the distribution of states in the band gap of PbI_2_ ($1\bar{1}0$)-perovskite (01$\bar{1}$) (Supplementary Fig. 15a), we observed that the state mostly originated from the *p* orbitals of Pb is  delocalized in the perovskite, which is still considered benign. The computed electronic structures and density of states (DOS) of the PbI_2_ (001)-perovskite (011) interface is shown in Fig. 3b. It can be seen that both the VBM and CBM are mainly contributed from the perovskite component, in which the VBM is composed of the antibonding I *p* orbitals and Pb *s* orbitals, while the CBM is composed of the *p* orbitals  of Pb and I with a majority nonbonding character (Supplementary Fig. 15b). Obviously, only some shallow states are localized around the band edge and no deep states exist in the band gap, demonstrating that this interface is generally harmless for the optoelectronic properties of relating device. Analogically, the interface of PbI_2_ ($\bar{1}1\bar{1}$)-perovskite (001) exhibits similar electronic structures and DOS (Fig. 3c and Supplementary Fig. 15c), implying that this interface can also be considered benign.

FA/Cs and I ions are intercalated between the layers of PbI_2_ around the interface, leading to an interfacial transition from PbI_2_ to perovskite driven by external energies. Also, interfaces can serve as sinks for migrating point defects, such as I interstitials and FA/Cs interstitials^26^. To illustrate the effect of point defect-interface interaction on the electronic structures of the interfaces, we considered the addition of I, FA/Cs, and Pb into the three interfaces, respectively. As shown in Fig. 3d–f, I interstitials are introduced into the three interfaces. It is found that the excess I at these interfaces all directly lead to localized trap states, mainly originating from the *p* orbitals of excess I atoms and neighboring I atoms (Supplementary Fig. 16). Similarly, Pb interstitials will also lead to the localized trap states in the band gap, which are mainly contributed by the *p* orbitals of Pb and I atoms around the defect (Supplementary Figs. 17, 18). For FA/Cs interstitials (Supplementary Figs. 19, 20), there are significant localized trap states originating from the *p* orbitals of Pb and I atoms close to defect exist in the band gap of PbI_2_ (001)-perovskite (011) (Supplementary Fig. 19b). In contrast, the states stemming from the FA/Cs interstitials at the interfaces of PbI_2_ ($1\bar{1}0$)-perovskite (01$\bar{1}$) and PbI_2_ ($\bar{1}1\bar{1}$)-perovskite (001) are closer to band edge and exhibit obviously delocalized characteristics (Supplementary Fig. 19a, c), demonstrating that FA/Cs interstitials at these two interfaces are generally harmless. Besides, owing to the well-known low formation energies of I vacancies at the surface or grain boundary, we also considered the I vacancies at the three interfaces (Supplementary Figs. 21, 22). Similar to I interstitials, I vacancies at the three interfaces all introduce localized trap states, which originated from the *p* orbitals of Pb and I atoms close to defects. Clearly, most of the common defects could introduce localized trap states in these interfaces, which will be detrimental to the performance of the device. Indeed, through the IGIA process, not only the expansion of intragrain impurities is prevented, but also the trap states formed at interfaces are suppressed by interface transition, demonstrating that the IGIA driven by external energies may be a promising strategy for defects elimination and optimizing the PCE.

**Fig. 4:**
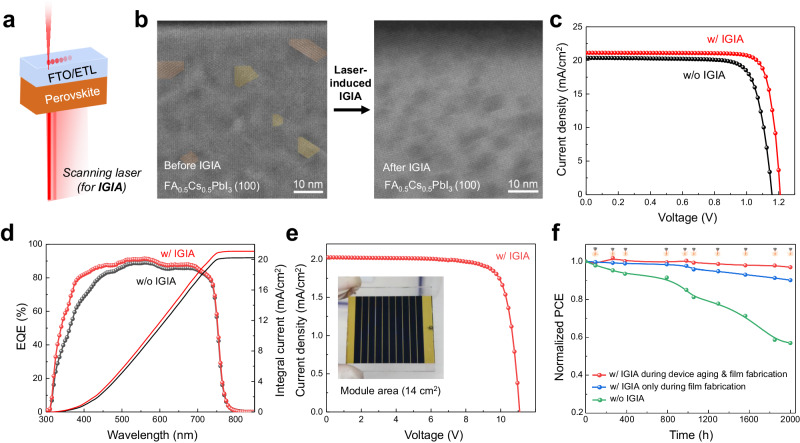
Leveraging intragrain impurity annihilation to improve perovskite solar cells. **a** Schematic illustration of using a scanning laser probe for in situ IGIA for PSCs; **b** High-magnification STEM-HAADF images of the intragrain microstructure before and after IGIA for the same grain region; orange and yellow regions represent PbI_2_ and non-PbI_2_ intermediate phases that exist in the neatly synthesized perovskite film. **c** Current-voltage (*J-V*) curves, and **d** IPCE spectra of PSCs before and after laser-induced IGIA; **e** *J-V* curve of perovskite solar module with active area of 14 cm^2^ with optimal laser treatment. **f** Stability tests results for PSCs with different laser treatment conditions. The green plots are referred to the device without IGIA. The blue plots are referred to the device with IGIA only during film fabrication. The red plots are referred to the device with IGIA during both film fabrication and device aging. For the red plots, the laser icon indicates the times when the additional laser treatments (during device aging) are performed.

The atomic-scale insights into the IGIA and its impacts on electronic structures have inspired us to leverage this phenomenon to improve PSCs. As schematically illustrated in Fig. 4a, we then used an optimized scanning laser to illuminate FA-Cs PSCs with a planar device configuration (FTO/TiO_2_-SnO_2_/perovskite/Spiro-OMeTAD/Au). The processing details are included in the Methods. We acquired STEM images of the PSC cross-section before and after laser treatment. Like the electron beam, scanning laser can also lead to effective IGIA in perovskites (Fig. 4b). In Fig. 4c, the current density-voltage curves of the FA_0.5_Cs_0.5_PbI_3_ PSC with and without laser-induced IGIA are compared. The treated PSC shows a PCE of 21.3%, with an open-circuit voltage (*V*_OC_) of 1.21 V, a short-circuit current density (*J*_SC_) of 21.13 mA/cm^2^, a fill factor (FF) of 0.833, demonstrating obvious improvement, as compared with the neatly fabricated PSC (PCE: 18.5%; *J*_SC_*:* 20.37 mA/cm^2^*; V*_OC_: 1.16 V; FF: 78.56%). The *J-V* hysteretic curves have been acquired (Supplementary Fig. 23), also showing improvement with the laser-induced IGIA. We also examined the effect of laser healing on the structure and optoelectronic properties of the FA-Cs perovskite film. According to a careful X-ray diffraction (XRD) analysis, we identified a weak characteristic peak of PbI_2_ in the pristine FA-Cs perovskite film, which disappears after the laser-induced IGIA process (Supplementary Fig. 24), consisting with the expected reduction in intragrain impurities coupled with IGIA. In addition, we performed the crystal structure refinement of the XRD patterns (Supplementary Fig. 25), revealing little impact of laser-induced lGIA on the lattice parameters and crystallite sizes in FA-Cs perovskite films. Besides, both top-view scanning electron microscopy (SEM) and atomic force microscopy (AFM) results (Supplementary Figs. 26–27) show little change in the perovskite film surface morphology after the laser-induced IGIA treatment, which implies that the PbI_2_ impurity healing may mostly occur inside the perovskite grains. The film after laser-induced IGIA also exhibits higher-intensity steady-state photoluminescent (PL) peaks and longer PL lifetime (Supplementary Figs. 28, 29 and Supplementary Table 1), confirming the decrease of trap density and thus nonradiative recombination^3^. Besides, the time-resolved photoluminescence (TRPL) spectra obtained from different sites of FA-Cs perovskite film confirm the uniformity of laser-induced IGIA treatment (Supplementary Fig. 29). The enhancement in the structure and properties of perovskites serves as a solid link between IGIA and device PCE increase. The integrated photocurrent densities (without IGIA: 20.22 mA cm^−^^2^; with IGIA: 21.08 mA cm^−2^) from the external quantum efficiency (EQE) spectra in Fig. 4d, are consistent with the extracted *J*_*SC*_ values from *J-V* curves. We also examined the statistics of device PCEs. Supplementary Fig. 30 presents a box chart distribution of PCEs based on 20 individual devices for each case (with and without laser-induced IGIA), demonstrating excellent reproducibility on the PCE improvement. Furthermore, proves the feasibility of laser-healing method for fabricating large-area, high-performance PSCs by presenting a 17.64% PCE, 14.00 cm^2^ solar module consisting 10 cells in series connection (Fig. 4e and Supplementary Fig. 31b), higher than that (16.13% PCE) of the solar module without the laser-induced IGIA treatment (Supplementary Fig. 31a). IGIA may be applied to other perovskite compositions. When we started with a FA_0.9_Cs_0.1_PbI_3_ PSC, laser-induced IGIA can deliver a PCE of 22.9%, with a *V*_*OC*_ of 1.15 V, a *J*_*SC*_ of 24.88 mA cm^−^^2^, an FF of 0.799, as compared with 21.9% for regular devices (Supplementary Fig. 32). Besides, with optimized processing conditions applied, the laser-induced IGIA can also be effective on other perovskite systems, such as MAPbI_3_, FAPbI_3_ and (FA_0.95_Cs_0.05_PbI_3_)_1-x_(MAPbBr_3_)_x_, as shown in Supplementary Figs. 33–35 and Supplementary Table 2. Finally, we examined the effect of laser-induced IGIA on the device stabilities of PSCs (Fig. 4f). Two different laser treatment methods were applied. One entails laser-induced IGIA only at the film fabrication stage, while the other involves additional laser treatments during the device storage time. After storing these devices for 2000 hours in the same conditions (25 ˚C, 10% RH in the ambient air), we have seen an improvement in the PCE retention, 57% and 90% for the PSC device without IGIA and with IGIA only at the film fabrication stage, respectively. Strikingly, once the device receives additional laser treatments during the device aging test, it can maintain 96% of the initial PCE after 2000 hours, demonstrating the feasibility of laser treatment for further recovering the performance of PSCs during storage. In addition, the device’s thermal stability and operational stability have also been tested for over 1000 h. As shown in Supplementary Fig. 36, both the thermal stability and operational stability are obviously improved, which can be attributed to the IGIA-coupled strain relaxation as further observed for laser-healed perovskite samples (Supplementary Fig. 37). It is worth noting that when the laser treatment time is too long (about 10 min), the device performance will also be adversely affected as demonstrated in Supplementary Fig. 38.

## Discussion

While all above STEM-DFT-device integrated studies have demonstrated the discovery of IGIA phenomena and its potential impact on device advancements, it is of vital importance to examine the generality of such discovery. Therefore, to further examine the feasibility of imposing IGIA effects on other perovskite systems, we applied in situ electron probe scanning to an MA-Cs perovskite specimen (MA_0.5_Cs_0.5_PbI_3_) which contains similar intragrain PbI_2_ nanoclusters (Supplementary Fig. 8). Similarly, these impurities can also be effectively healed to perovskite phase (Supplementary Figs. 39, 40), consistent with our observations in FA-Cs perovskite. This result demonstrates the potential generality of external stimuli-driven IGIA in the broad perovskite composition space. Furthermore, to rule out the possibility that IGIA results from the incomplete perovskite crystallization, we carry out in situ observation on an over-annealed FA-Cs perovskite specimen (Supplementary Fig. 3b), and the intragrain PbI_2_ nanoclusters can still be annihilated (Supplementary Fig. 41). We note that by examining the IGIA reaction stoichiometry, excess FA/Cs cations and halide anions are necessary, which may be derived from the solution processing additive (such as FACl in this work). In the pristine state of as-synthesized perovskite films, these excess ions may be dispersed within the grain boundary regions, which can be activated by external stimuli, thus migrating into the reaction fronts of PbI_2_/perovskite transformation. To attest to this, we prepare the (FA,Cs)PbI_3_ and (MA,Cs)PbI_3_ specimens without the addition of FACl and MACl. As a result, continuous electron probe scanning drives no variation or even gradual expansion of the existing intragrain PbI_2_ impurity nanoclusters rather than shrinkage and annihilation (Supplementary Figs. 42, 43). Besides, referring to the PSC device fabricated from (FA,Cs)PbI_3_ without the addition of FACl, the same laser treatment is found less effective in improving the device stability and PCE recovery (Supplementary Fig. 44). This implies the possible role of FACl addition in perovskite synthesis on the IGIA effect, which is interesting for further exploration. Moreover, the influence of electron dose rate on the IGIA process is also examined. As shown in Supplementary Fig. 45, using a smaller 1 pA electron beam current (corresponds to 105 e·Å^−^^2^/s dose rate), a similar IGIA phenomenon can be observed but a significantly longer irradiation time is required. Therefore, the overall dose for healing the small-size PbI_2_ nanoclusters exhibits a similar magnitude which is likely to be independent of the dose rate (Supplementary Figs. 39, 41, 45). However, if a significantly larger electron beam current up to 10 pA is adopted, the perovskite structure framework tends to collapse in the irradiated region rather than heal the intragrain PbI_2_ nanoclusters (Supplementary Fig. 46).

In closing, we leverage the atomic-scale in situ STEM methodology to investigate the phase and structural dynamics in PSCs, and we visualize various crystallographic transformation modes of intragrain PbI_2_ impurity phases being healed to perovskites with atomic-scale evidence, strikingly in contrast to our intuitive thinking that perovskites always tend to degrade under external stimuli. We further demonstrate that such fundamental findings are translatable to accelerate innovations in processing and treatment methods for achieving more efficient and stable solar cells. We envision that more advanced in situ TEM studies, incorporating atmosphere, temperature, and even light conditions^39^, will generate more translatable fundamental discoveries for the advancement of multi-functional perovskite optoelectronics.

## Methods

### MHP precursor solution and film synthesis

The FA-Cs mixed MHP (FA_0.5_Cs_0.5_PbI_3_, MA_0.5_Cs_0.5_PbI_3_) thin films are prepared according to the method reported earlier^40^. First, 1.33 M MHP precursor solution of FA_0.5_Cs_0.5_PbI_3_ (MA_0.5_Cs_0.5_PbI_3_) was prepared by dissolving FAI (MAI), CsI, FACl (MACl), and PbI_2_ with ratios of 0.5:0.5:0.5:1 in dimethyl sulfoxide (DMSO). The precursor solutions were stirred at 65 °C for 6 h to be ready for use. Then the perovskite solution was spin-coated in a two-step at 1000 rpm and 5000 rpm for 10 s and 60 s, respectively. During the second step, 380 × 2 μl ethyl acetate was drop-casted quickly at the 20 and 40 s of the second step. The perovskite film was then heated at 100 ˚C for 2 min in the glove box and 140 ˚C for 60 min in ambient condition (RH ≈ 30–40%) to form the FA-Cs mixed MHP thin film. This annealing time is optimized by XRD measurement as shown in Supplementary Fig. 47. Besides, further SEM images (Supplementary Figs. 48, 49), UV-vis spectra (Supplementary Fig. 50), and performance test (Supplementary Fig. 51) also confirm 60 min should be the optimal annealing time. The crystallization of FA-Cs MHP is completed after 60 min annealing with less PbI_2_ formed than 90 min annealing, this has also been confirmed by STEM observation (Supplementary Fig. 3). As for the MA_0.5_Cs_0.5_PbI_3_ thin films, the annealing temperature is 100 ˚C for 60 min in ambient condition (RH ≈ 30–40%).

### Device fabrication

Fluorine-doped tin oxide (FTO)-coated glass (2.20 mm, 7Ω sq^−^^1^) was used as the substrate for the devices. The compact TiO_2_ layer (~10 nm) was deposited by atomic layer deposition (ALD) for 200 cycles and annealed at 500 ˚C for 30 min in ambient air. For ALD TiO_2_ deposition, titanium (IV) isopropoxide (TTIP) and H_2_O as Ti and O sources, respectively. The TTIP precursor was held at 75 ˚C. Pulse/exposure/purge times of 1 s/8 s/25 s was used for the TTIP and 0.1 s/8 s/25 s for H_2_O precursor, and the deposition temperature was set to 120 ˚C. On top of the c-TiO_2_ layer, SnO_x_-Cl layer was deposited by spin-coating at a rate of 3000 rpm for 30 s from an aged SnCl_4_ aqueous solution (1:75 with deionized water by volume), followed by a sintering heat-treatment of 200 ˚C for 30 min in air and then transferred to the glove box for device fabrication. The perovskite solution was spin-coated in a two-step at 1000 rpm and 5000 rpm for 10 s and 60 s, respectively. During the second step, 380 × 2 μl ethyl acetate was drop-casted quickly at the 20 and 40 s of the second step. The perovskite film was then heated at 100 ˚C for 2 min in the glove box and 140 ˚C for 60 min in ambient condition (RH≈30–40%). Subsequently, the films were treated by laser annealing process. For the surface passivation, 1 mg ml^−^^1^ of Methoxy-Phenethylammonium iodide (MeO-PEAI) solution in isopropanol was spin-coated on these perovskite films at 4000 rpm for 30 s. The spiro-OMeTAD chlorobenzene solution (72.3 mg ml^−^^1^) with 28.8 μl 4-tert-butylpyridine (96%, Aldrich–Sigma) and 17.5 μl lithium bis(trifluoro-methanesulfonyl) imide (Li-TSFI, Aldrich–Sigma) solution (520 mg Li-TSFI (98%)) in 1 ml acetonitrile (99.8%, Aldrich–Sigma) was spin-coated on top of the perovskite film at 3000 rpm for 30 s. The devices were put into a dry-air box (RH < 5%) for 12 h. Finally, 80 nm thick Au electrode was thermally evaporated.

For fabricating modules, P1 etching process was pre-patterned on FTO glass (5 cm × 5 cm) with a 1064 nm fiber laser (Han’s laser). The laser power ratio, laser duty cycle, and laser frequency were 30%, 5%, and 50 kHz, respectively. Then, patterned FTO substrates were cleaned and treated by UV Ozone Cleaner (Ossila) for 15 min. The TiO_2_/SnO_2_, MeO-PEAI, and Spiro-OMeTAD layers were prepared with the same procedure as presented above. The large-size perovskite film was prepared the same as above. The perovskite solution was spin-coated in a two-step at 1000 rpm and 4000 rpm for 10 s and 60 s, respectively. During the second step, 600 × 2 μl ethyl acetate was drop-casted quickly at the 20 and 40 s of the second step. The perovskite film was then heated at 100 ˚C for 2 min in the glove box and 140 ˚C for 60 min at ambient condition (RH≈30–40%). Subsequently, the film was treated by a laser annealing process. For P2 etching process, the laser used was a 532 nm laser with a laser power ratio of 65%, laser duty cycle of 5%, and laser frequency of 100 kHz. 80 nm thick Au electrodes were thermally evaporated under vacuum to complete the modules fabrication. Finally, P3 etching used the same laser as P2 with a laser power ratio of 50%, laser duty cycle of 5%, and laser frequency of 100 kHz. P4 is an etching procedure for cleaning the edge of the modules, the laser used in P4 is the same as P1 with a laser power ratio of 40%, laser duty cycle of 10%, and laser frequency of 100 kHz.

### Laser-induced IGIA treatment for PSCs

The film is scanned (estimated scanning speed is 2 cm/s) and irradiated from the glass side for 5 min at a height of about 70 cm from the film with a laser (XINRUI, laser power is 500 mW, laser wavelength is 405 nm, laser spot size is 0.3 mm and the laser irradiation area is around 10 mm^2^) after the annealing process. The substrate temperature during laser treatment is 22.8 °C measured by an infrared thermometer.

### Materials characterization

XRD spectra were measured by Ultima IV of Rigaku with Cu Kα radiation (1.5406 Å) from 10° to 40° with 0.01° step of scan, speed of scan 10° min^−^^1^(Voltage 45 kV, Current 200 mA). PbI_2_ calibration reference standard PDF card (07-0235). The UV-Vis absorbance spectra were measured by QE Pro (Ocean Optics). Steady PL spectra were recorded on QE Pro excited at 460 nm. EQE measurement was calculated using certified incident photon to current conversion efficiency equipment from Enlitech (QE-R). Time-resolved photoluminescence (TRPL) experiments were performed by Steady State and Transient State Fluorescence Spectrometer (HORIBA Nanolog). The testing conditions as the films were photoexcited at 440 nm pulse width~60 ps, 3.0 mW/pulse, and emission were collected on the surface side of the film (perovskite/ glass substrate) at 760 nm with 5 nm slit size. The lifetime was calculated by bi-exponential fitting with the Expdec2 function Y = A_1_exp(-t/τ_1_) + A_2_exp(-t/τ_2_).

### PSC testing

For determining PCEs, *J*-*V* curves of the as-fabricated PSCs were measured using a SourceMeter (Keithley 2400) under simulated one-sun AM 1.5 G 100 mW cm^−^^2^ intensity (LED) with a scan rate of 200 mV/s (the voltage step is 20 mV with no delay time) from forward scanning directions in air condition around 25 ˚C. The typical active area of PSCs is 0.09 cm^2^ defined by a metal mask. The intensity of one-sun AM 1.5 G illumination was calibrated using a Si-reference cell certified by the National Renewable Energy Laboratory. For the device stability, the devices were stored in a dry-air box with a relative humidity of 10%. For the stability tests, all PSCs were without encapsulation. For storage stability, the devices were stored in a dark environment with a humidity of 10–30%, and the photovoltaic performance of PSCs was measured at intervals. The thermal stability was carried out by repeating the *J-V* test at intervals for the devices heated at a fixed temperature of 85 °C in a nitrogen glove box. The operational stability was performed using a stability setup (LC Auto-Test 24, Shenzhen Lancheng Technology Co., Ltd.), tested under continuous light illumination and maximum power point tracking (controlled and monitored to be 15 ˚C). The light source consisted of an array of white LEDs powered by a constant current. Equivalent sun intensities were calibrated using a calibrated Si-reference cell. During aging, the device is connected with a 100 Ohm load resistance. The PSCs were masked and placed inside a sample holder purged with continuous N_2_ flow. *J-V* curves with reverse voltage scans were recorded every 6 h during the whole operational test.

### STEM methods

The cross-sectional TEM specimens of PSCs were prepared by a dual-beam focused ion beam (FIB) nanofabrication platform (Helios 5 CX, Thermofisher). A protection layer was first deposited on the top surface of the devices by electron deposition of Pt, followed by etching the surrounding area to form the specimen lamella. The operation voltage of the gallium ion beam is 30 kV and the working current is 0.1–24 nA for lamella processing. The lamella was then lifted out from the substrate and in situ transferred to a TEM half grid inside the FIB chamber. The observation area of lamella was thinned to less than 100 nm with 40–790 pA gallium ion beam. To minimize the damage induced by ion implantation to sample lamella, a fine milling and polishing process was adopted using a gallium ion beam with accelerating voltage down to 1 kV and 72 pA working current to remove the surface amorphous layer. After FIB preparation and polishing procedures, the as-prepared cross-sectional PSC specimens were transferred to a high vacuum sputter coater for protection layer deposition. Amorphous carbon layers with a thickness of 10 nm were coated on both sides of the cross-section specimen using pulsed carbon evaporation at 8 × 10^−^^5^ mbar.

STEM observations of the device cross-section specimens were carried out in an aberration-corrected STEM microscope (Titan G2 60–300 and Spectra 300, Thermofisher equipped with a field emission gun) with 300 kV electron beam accelerating voltage. The beam current of the electron probe was reduced to 5 pA to minimize the damage to MHP frameworks during atomic resolution imaging. The probe convergence angle was 24.5 mrad, and the angular range of the HAADF detector was from 79.5 to 200 mrad. The dwell time of each pixel during STEM-HAADF image acquisition is 6 μs, and the size of all STEM-HAADF images in this work is 2048 × 2048 pixel^2^. For the typical high-resolution STEM-HAADF images illustrated in this work, the frame size is 34.5 × 34.5 nm^2^. For in situ electron probe continuous scan of regions of interest (ROI) in our experiment, we used 5 pA beam current and 1 μs dwell time for each pixel with scanning pixel size set as 1024 × 1024 pixel^2^. The scanning frame size is also kept at 24.4 × 24.4 nm^2^. The average dose rate can be calculated as 525 e·Å^−^^2^/s.

Plan-view STEM studies on the same FA-Cs perovskite thin film specimen based on the liquid-phase release-and-transfer process reported earlier by us^41^ were also performed to confirm the intragrain nature of observed impurity nanoclusters in cross-sectional STEM studies.

### Computational methods

All ab initio computations for the periodic systems are performed based on density-functional theory (DFT) methods as implemented in the Vienna ab initio simulation package (VASP 5.4)^42^. The projector-augmented wave (PAW) method with the generalized gradient approximation of Perdew–Burke–Ernzerhof (PBEsol) exchange-correlation functional was adopted. The Heyd–Scuseria–Ernzerhof (HSE06, α = 0.43) hybrid functional was applied to compute the electronic structure more accurately. The SOC was considered in the calculation due to the inclusion of the heavy metal element Pb. The cut-off energy was set to be 520 eV for structure optimization, and 400 eV for electronic structure calculations. The convergence thresholds for the energy difference and the force were set to be 1 × 10^−4^ eV and 4 × 10^−2^ eV/Å, respectively. The Monkhorst-Pack k-point mesh of 1 × 1 × 1 was used for Brillouin zone integration. The supercells of PbI_2_ ($\bar{1}1\bar{1}$)-perovskite (001), PbI_2_ (001)-perovskite (011) and PbI_2_ ($1\bar{1}0$)-perovskite (01$\bar{1}$) are 12.5 Å × 12.5 Å × 50.7 Å, 15.3 Å × 8.8 Å × 41.3 Å and 8.8 Å × 15.3 Å × 48.5 Å, respectively.

### Reporting summary

Further information on research design is available in the Nature Portfolio Reporting Summary linked to this article.

### Supplementary information

Supplementary Information

Reporting Summary

## Data Availability

The authors declare that data supporting the findings of this study are available within the paper and its Supplementary Information files. The experiment raw data of this study are available from the corresponding author upon request.
