# Supplementary material for: Intragrain impurity annihilation for highly efficient and stable perovskite solar cells
Source: Nat Commun. 2024 Mar 14;15:2329. doi: 10.1038/s41467-024-46588-y (PMC10940583; doi:10.1038/s41467-024-46588-y)
Supplement: Supplementary file 1 — Supplementary Information [file 41467_2024_46588_MOESM1_ESM.pdf]

Supplementary Information

Intragrain impurity annihilation for highly efficient and stable perovskite solar cells

Songhua Cai[1, †,*], Zhipeng Li[2, †], Tanghao Liu[3], Yalan Zhang[4], Peng Wang[5], Ming-Gang Ju[6,*], Shuping Pang[2,*], Shu Ping Lau[1], Xiao Cheng Zeng[7], Yuanyuan Zhou[4*]

[1] Department of Applied Physics, The Hong Kong Polytechnic University, Kowloon, Hong Kong SAR, China

[2] Qingdao Institute of Bioenergy & Bioprocess Technology, Chinese Academy of Sciences, Qingdao, Shandong 266101, China

[3] Department of Physics, Hong Kong Baptist University, Kowloon, Hong Kong SAR, China

[4] Department of Chemical and Biological Engineering, The Hong Kong University of Science and Technology, Clear Water Bay Kowloon, Hong Kong SAR, China

[5] Department of Physics, University of Warwick, Coventry, CV4 7AL, United Kingdom

[6] School of Physics, Southeast University, Nanjing 211189, China

[7] Department of Materials Science and Engineering, City University of Hong Kong, Kowloon, Hong Kong SAR, China

[†] These authors contributed equally: Songhua Cai and Zhipeng Li.

*Correspondence should be addressed to: yyzhou@ust.hk; juming@seu.edu.cn; pangsp@qibebt.ac.cn; songhua.cai@polyu.edu.hk

This file includes:

Supplementary Figures 1 to 51

Supplementary Tables 1 to 2.

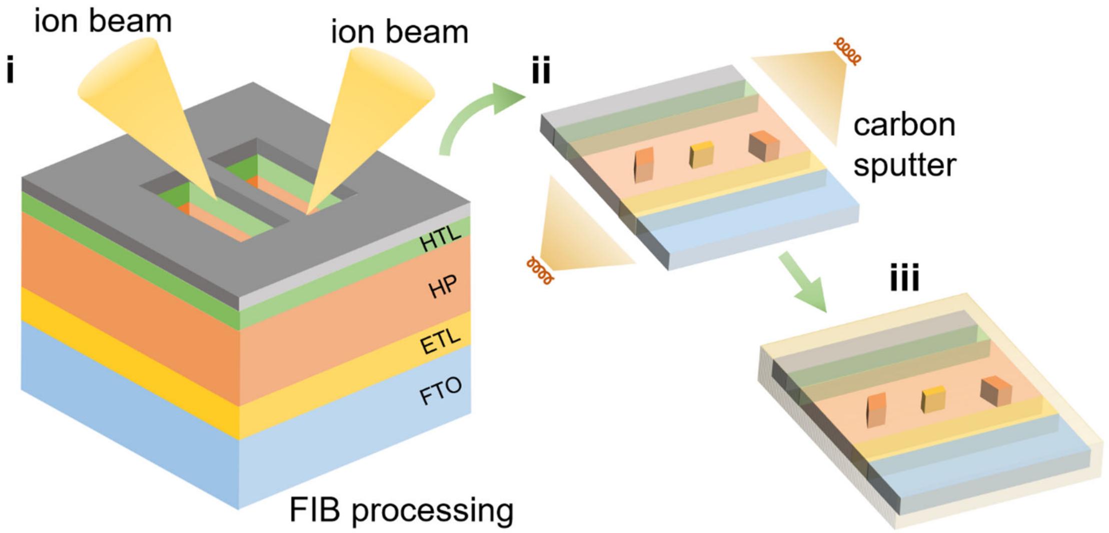

Supplementary Fig. 1 Workflow of the sample preparation for our *in situ* STEM study. (i) The device sample lamella was first fabricated using FIB, (ii) then a 10 nm amorphous carbon coating was deposited from both sides, and finally, (iii) the sample lamella was wholy wraped and stablized.

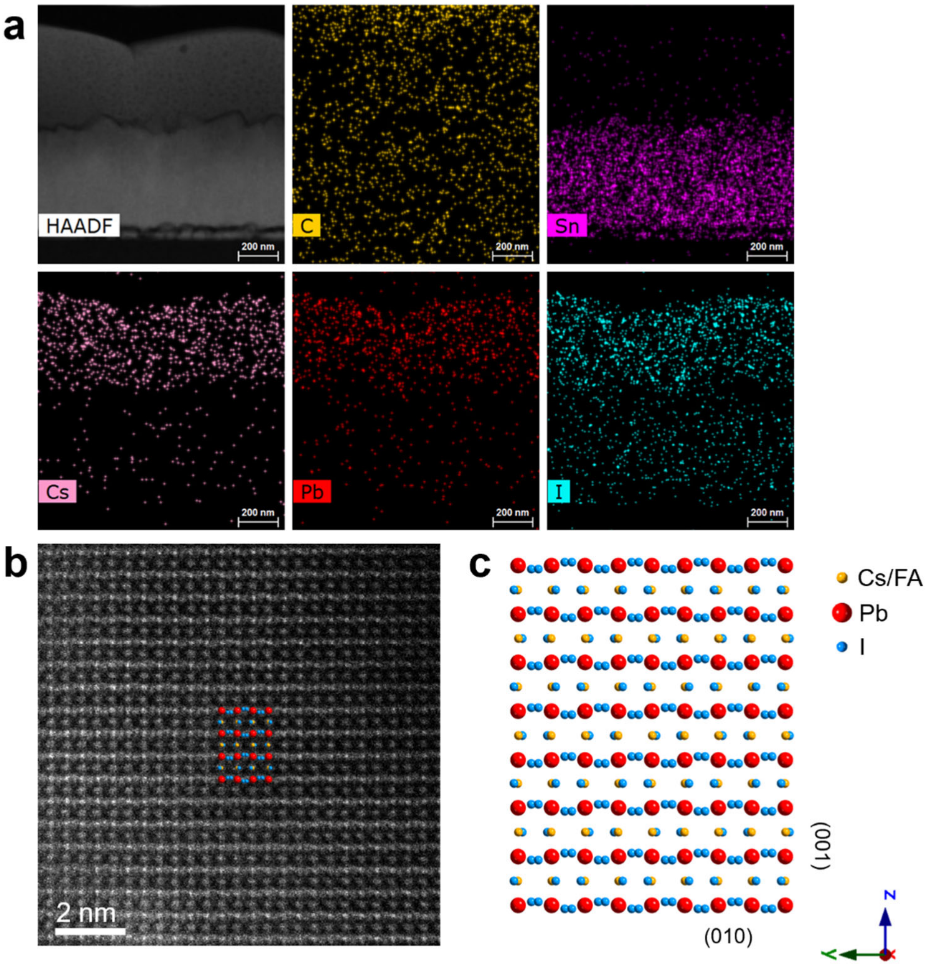

Supplementary Fig. 2 STEM-EDS mapping and atomic structure of the orthorhombic perovskite grain. a, STEM-EDS mapping of the FIB-fabricated PSC device cross-sectional specimen. **b,** Atomic resolution STEM-HAADF image of the orthorhombic perovskite $FA_{0.5}Cs_{0.5}PbI_3$ grain projected along $[100]_o$ direction. **c,** Atomic model of the orthorhombic perovskite (space group *Pnma*) viewed from $[100]_o$ direction, resolved according to the STEM-HAADF observation.

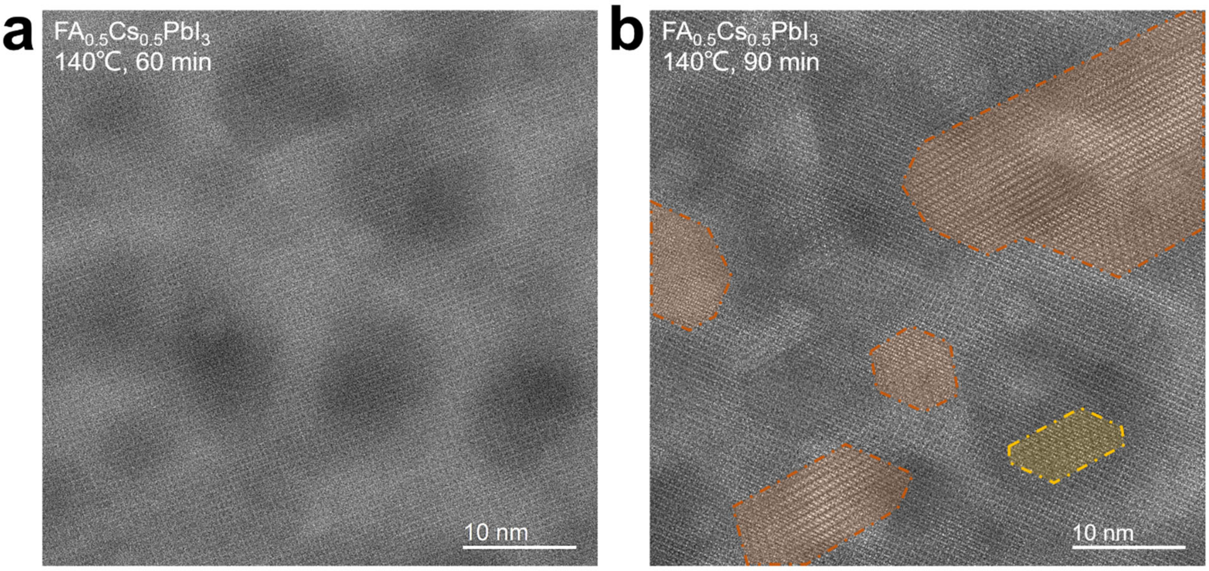

Supplementary Fig. 3 The effect of annealing time on the distribution of perovskite intragrain impurity nanoclusters. a, High-resolution cross-sectional STEM-HAADF image of the $FA_{0.5}Cs_{0.5}PbI_3$ film prepared by the annealing condition (140 °C, 60 min), showing a very low densities of intragrain impurities, respectively. **b,** High-resolution cross-sectional STEM-HAADF image of the $FA_{0.5}Cs_{0.5}PbI_3$ film prepared by the annealing condition (140 °C, 90 min), showing a high density of intragrain impurities.

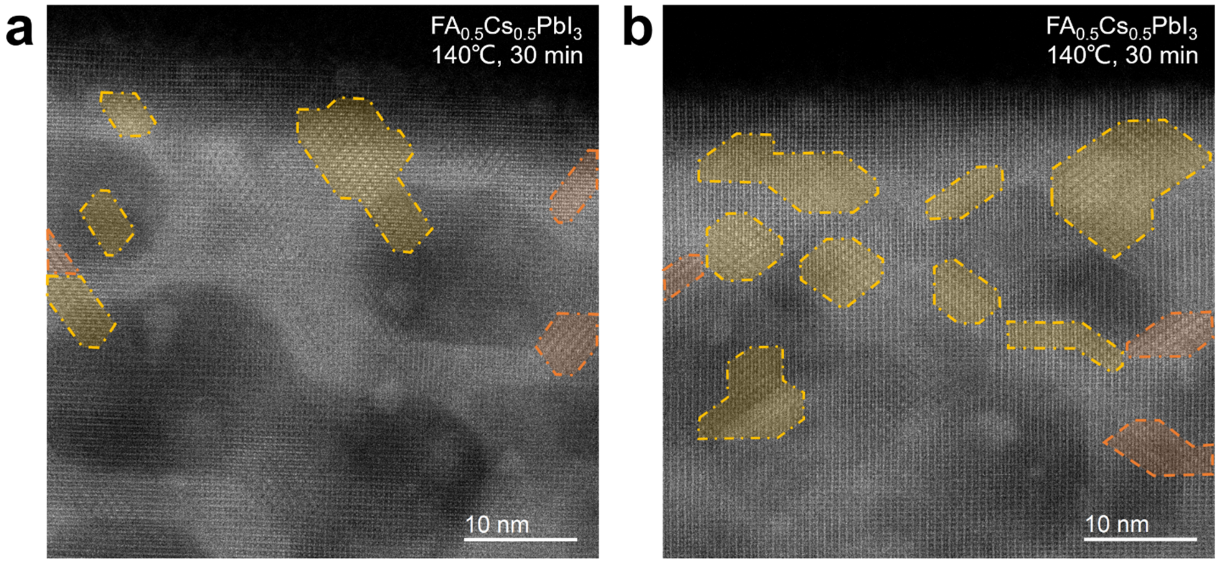

Supplementary Fig. 4 The effect of short annealing time on the distribution of perovskite intragrain impurity nanoclusters. a, b, High-resolution cross-sectional STEM-HAADF image of the $FA_{0.5}Cs_{0.5}PbI_3$ film prepared by the annealing condition (140 °C, 30 min), showing a high density of intragrain impurities.

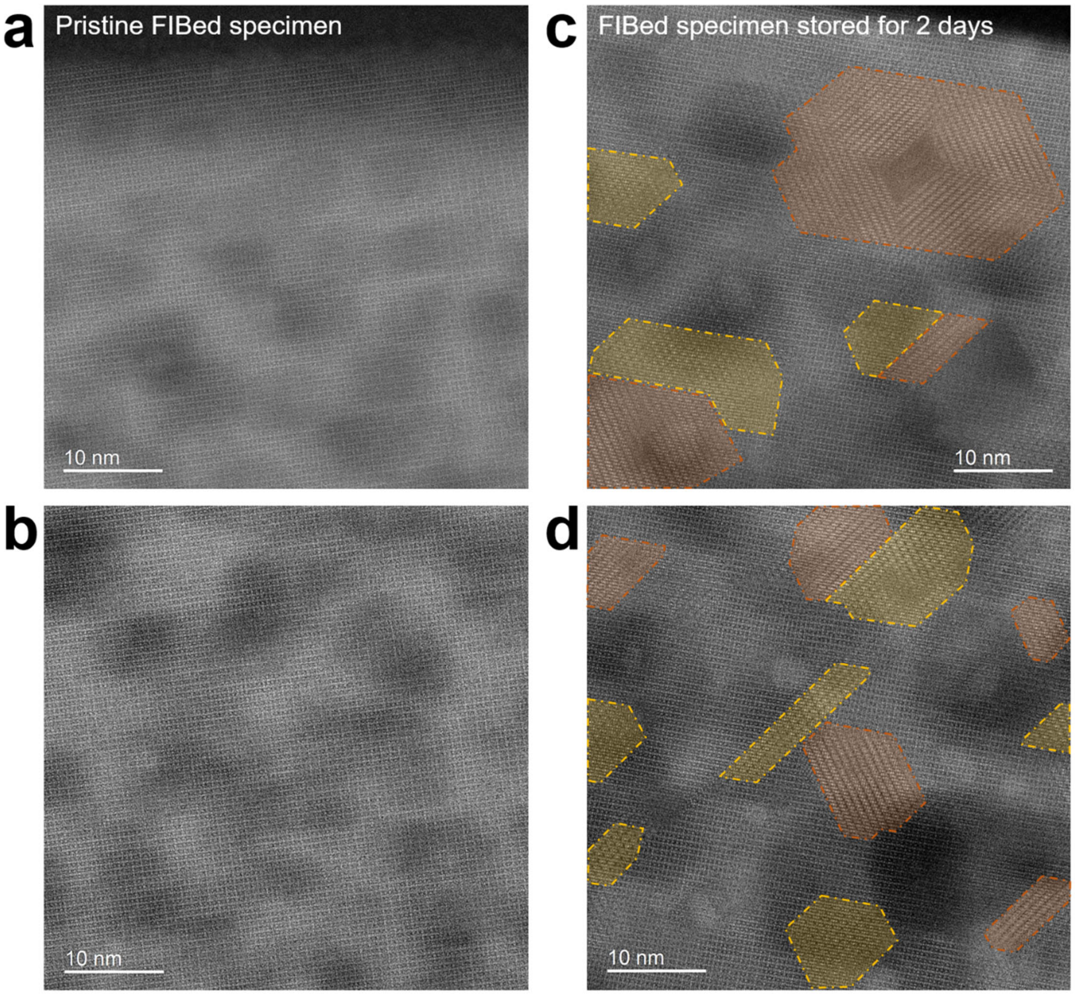

Supplementary Fig. 5 The influence of reservation to perovskite intragrain impurities. a, b, High-resolution STEM-HAADF images of the cross-sectional $FA_{0.5}Cs_{0.5}PbI_3$ specimen observed immediately after the FIB processing, taken from the perovskite grain surface and inner regions, respectively. The density of intragrain impurities at this stage is low. **c, d,** High-resolution STEM-HAADF images of the cross-sectional $FA_{0.5}Cs_{0.5}PbI_3$ specimen after 2-day storage in the vacuum package. A significant amount of intragrain impurities can be observed at this stage.

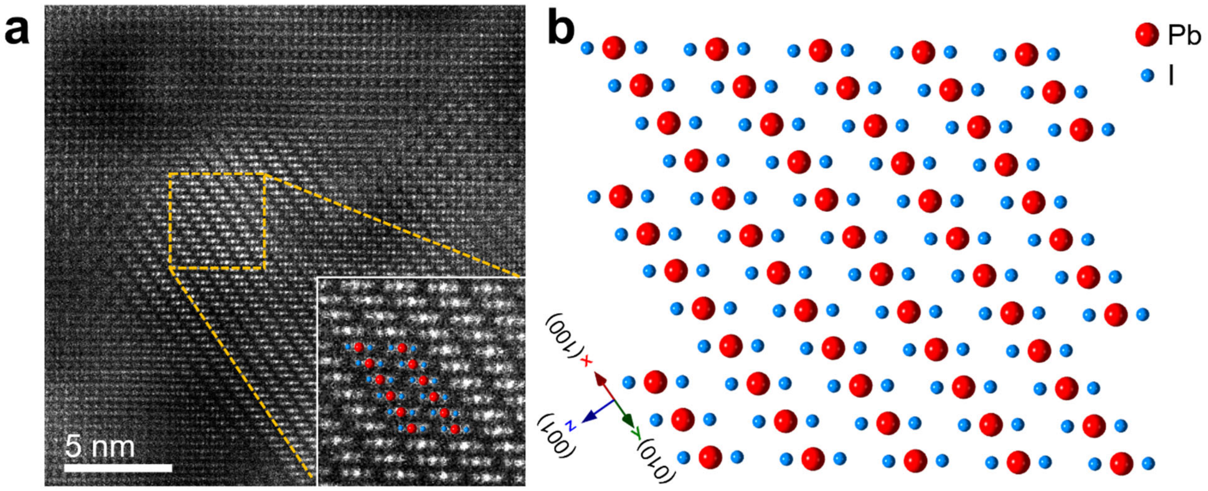

Supplementary Fig. 6 Atomic structure of the PbI_2 nanocluster in perovskite grain. a, Atomic resolution STEM-HAADF image of the orthorhombic perovskite $FA_{0.5}Cs_{0.5}PbI_3$ grain containing a PbI_2 nanocluster projected along $[110]_o$ direction. Inset is the enlarged image of the selected region, showing the detailed PbI_2 atomic structure that is consistent with the standard structure model. **b,** Atomic model of PbI_2 viewed from $[110]_o$ direction.

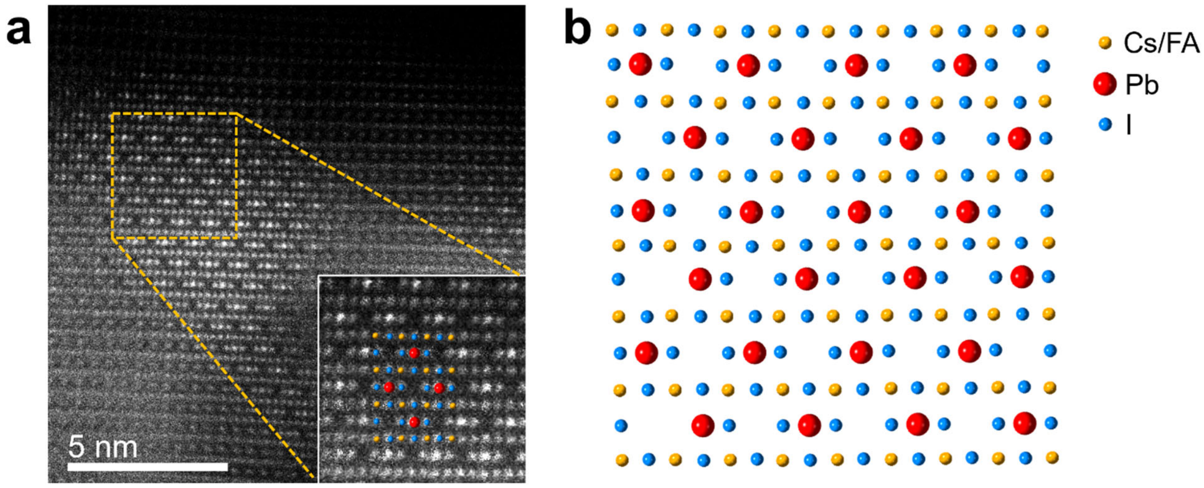

Supplementary Fig. 7 Possible atomic structure of the non-PbI_2 intermediate phase nanocluster in the perovskite grain. a, Atomic-resolution STEM-HAADF image of an orthorhombic perovskite $FA_{0.5}Cs_{0.5}PbI_3$ grain (projected along $[100]_o$ direction) contains a possibly non-PbI_2 intermediate phase nanocluster. Inset is the enlarged image of the selected region, showing the detailed atomic structure. **b,** Possible atomic model of the non-PbI_2 intermediate phase resolved according to the STEM-HAADF image.

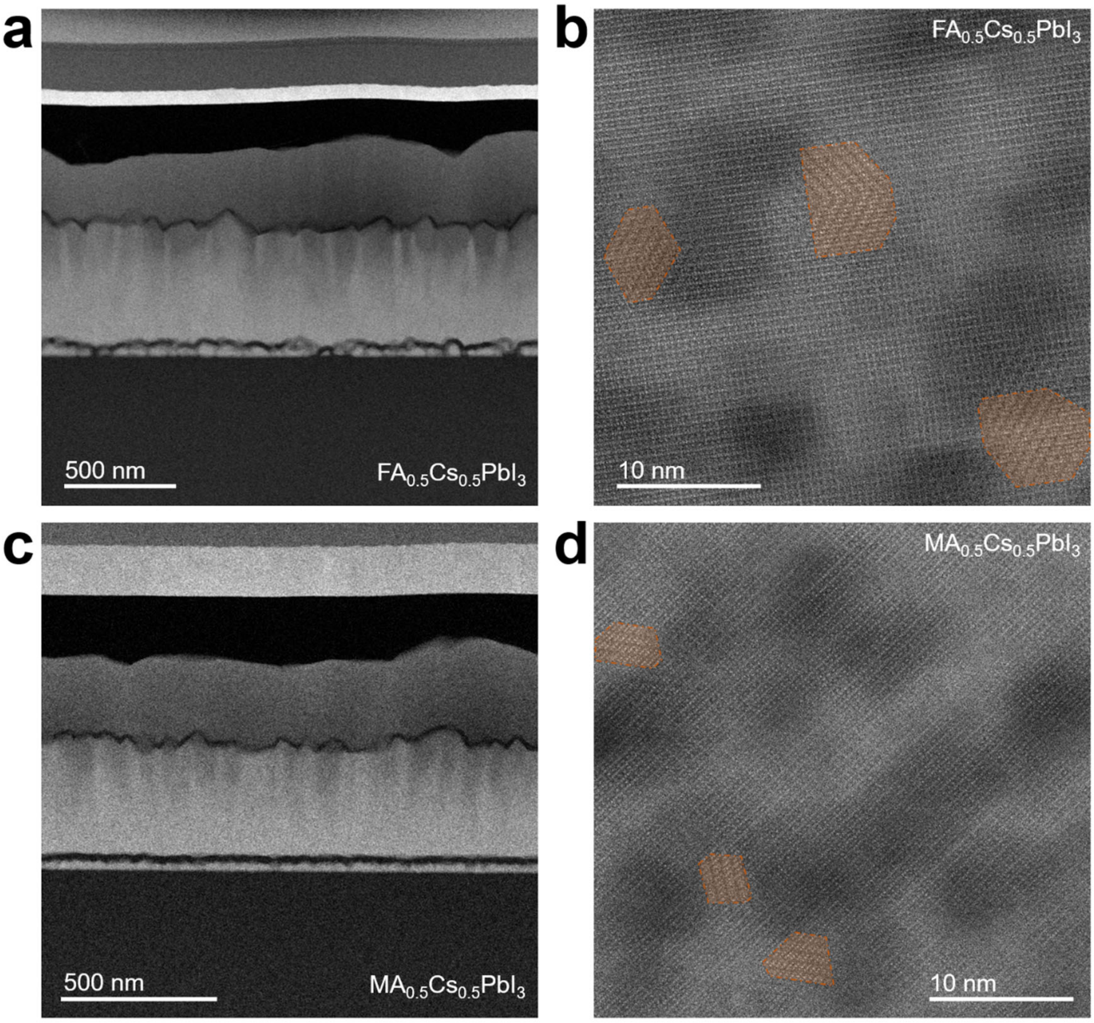

Supplementary Fig. 8 Intragrain impurities in different perovskite systems. A, Low-magnification STEM-HAADF image of the cross-sectional $FA_{0.5}Cs_{0.5}PbI_3$ PSC device specimen, showing no obvious FIB-caused damage in the perovskite layer. **b,** High-resolution STEM-HAADF image show a small amount of intragrain PbI_2 impurity nanoclusters. **c,** Low-magnification STEM-HAADF image of cross-sectional $MA_{0.5}Cs_{0.5}PbI_3$ device specimen, indicating no obvious FIB-caused damage in the perovskite layer. **d,** High-resolution STEM-HAADF image shows the existence of a similar distribution of intragrain PbI_2 nanoclusters.

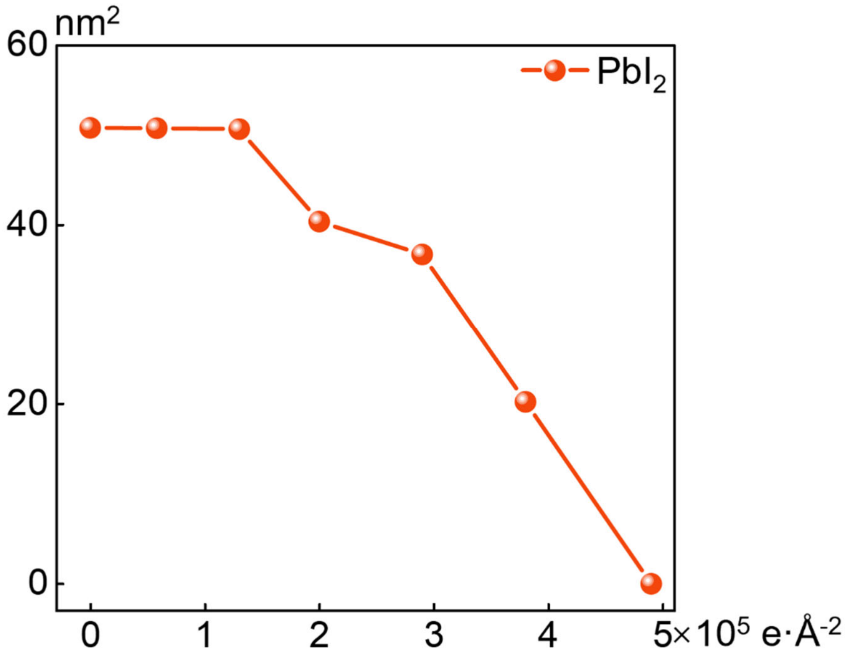

Supplementary Fig. 9 The size shrinkage of the PbI_2 nanocluster with respect to the accumulated electron dose in Fig. 1d.

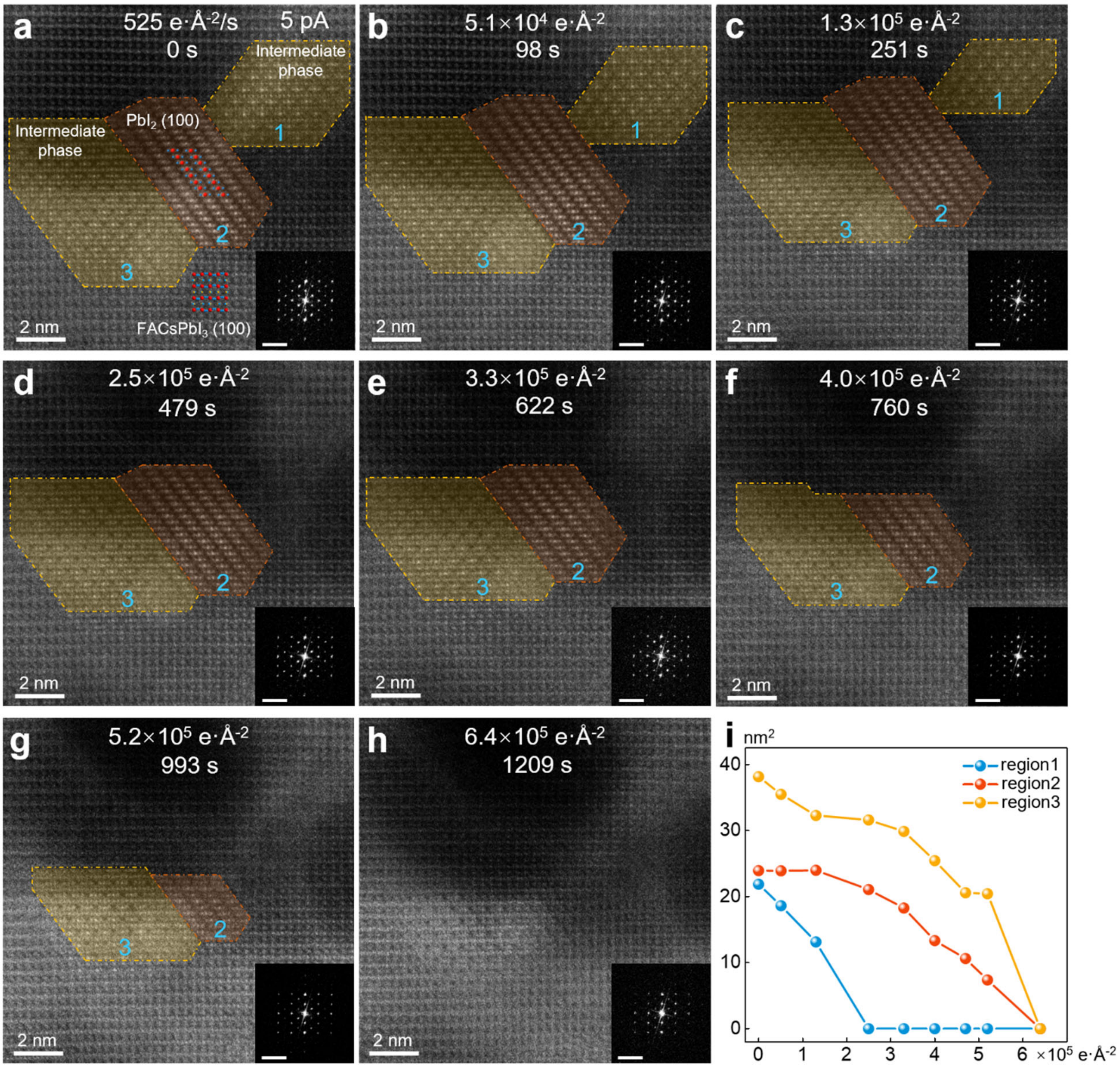

Supplementary Fig. 10 Atomic level *in situ* observation of mixed phase-healing process. a, Atomic resolution STEM-HAADF image of orthorhombic (FA,Cs)PbI_3 nano-region projected along [100]$_o$ direction contains both PbI_2 (marked by orange region and dashed lines) and non-PbI_2 impurity phase (marked by yellow regions and dashed lines) nanoclusters. **b-h,** The *in situ* phase-healing process with continuous electron probe scanning. Insets are corresponding FFT patterns of each STEM image. Scale bar: 4 1/nm. **i,** The area shrinkage of regions 1-3 with respect to the accumulated electron dose.

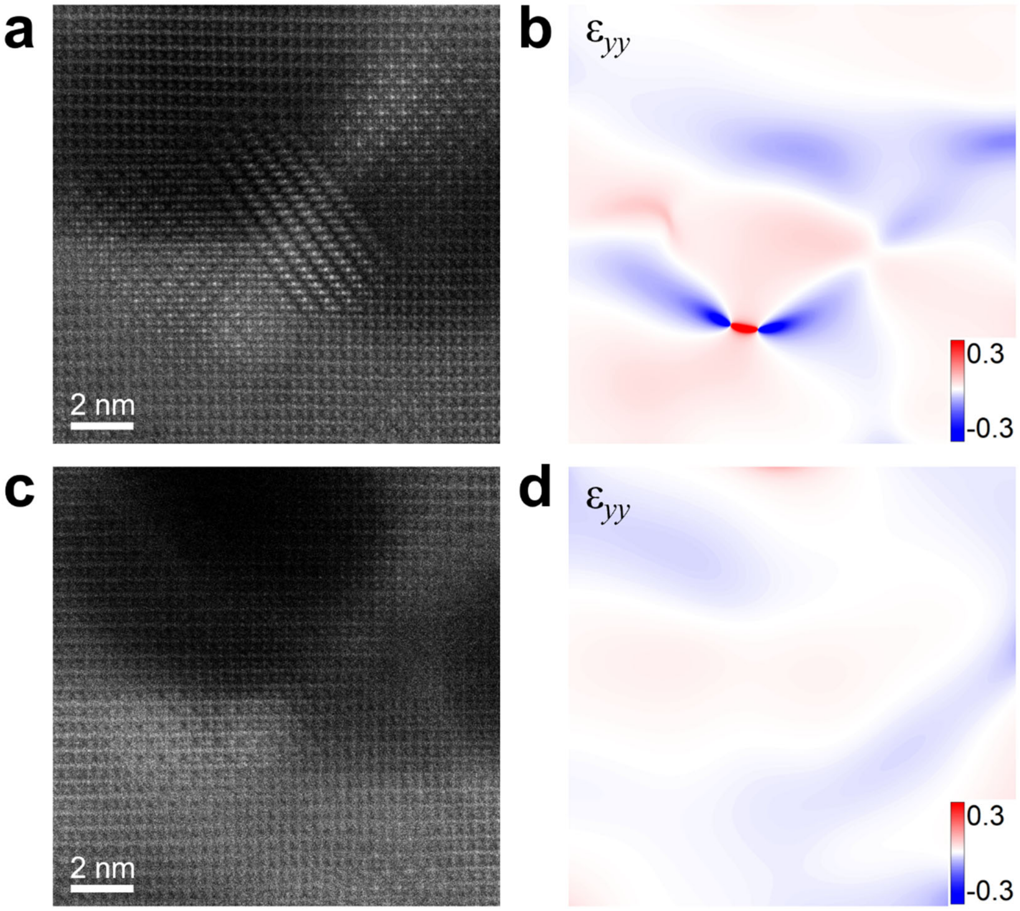

Supplementary Fig. 11 Perovskite intragrain strain relaxation by phase-healing. a, Atomic-resolution STEM-HAADF image of a perovskite grain contains both PbI_2 nanocluster and non-PbI_2 impurity phase, similar to that in Supplementary Fig. 6a. **b,** GPA mapping of out-of-plane strain (ε_{yy}) distribution in **a**, showing the PbI_2 nanocluster and non-PbI_2 impurity phase can induce uneven intragrain strain distribution. **c,** STEM-HAADF image after IGIA, similar to that in Supplementary Fig. 6h. **d,** GPA mapping of out-of-plane strain (ε_{yy}) distribution in **c**, showing the intragrain strain is relaxed.

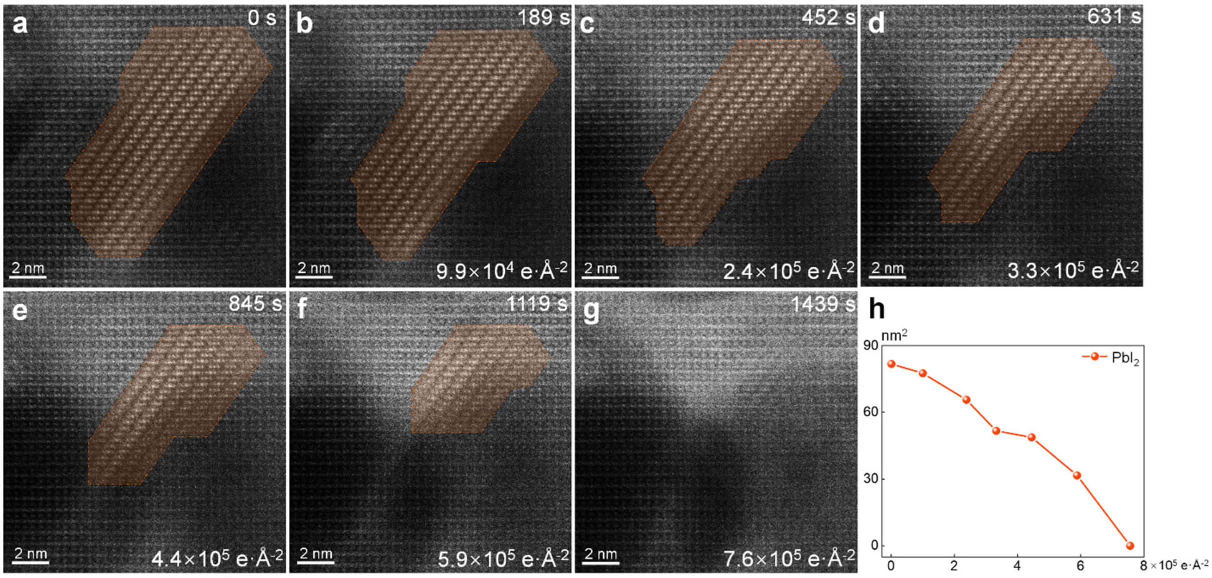

Supplementary Fig. 12 Transformation of a medium-size PbI_2 impurity nanocluster to perovskite. a, Atomic resolution STEM-HAADF image of orthorhombic $FA_{0.5}Cs_{0.5}PbI_3$ nano-region projected along $[100]_o$ direction contains a relatively large PbI_2 nanocluster with a projected size of ~80 nm^2 (marked with the orange coloring). **b-g,** The in-situ recorded transformation process of the PbI_2 nanocluster under a continuous electron probe scanning. **h,** The size shrinkage of the PbI_2 nanocluster with respect to the accumulated electron dose.

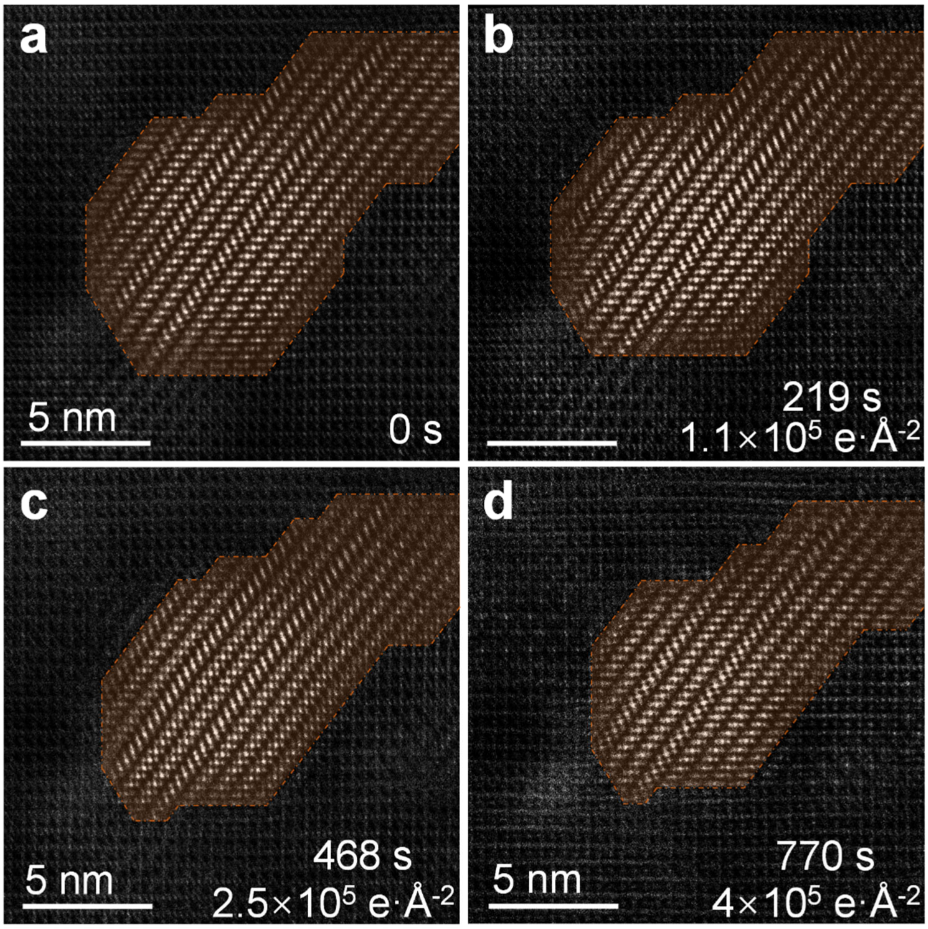

Supplementary Fig. 13 The beginning stage of the transformation from a large-size PbI_2 nanocluster to perovskite. a, Atomic resolution STEM-HAADF image of orthorhombic $FA_{0.5}Cs_{0.5}PbI_3$ nano-region projected along $[100]_o$ direction contains a much larger PbI_2 nanocluster with a size exceeding 120 nm^2 (marked by the orange coloring). **b-d,** The *in situ* recorded transformation process of the PbI_2 nanocluster with continuous electron probe scanning.

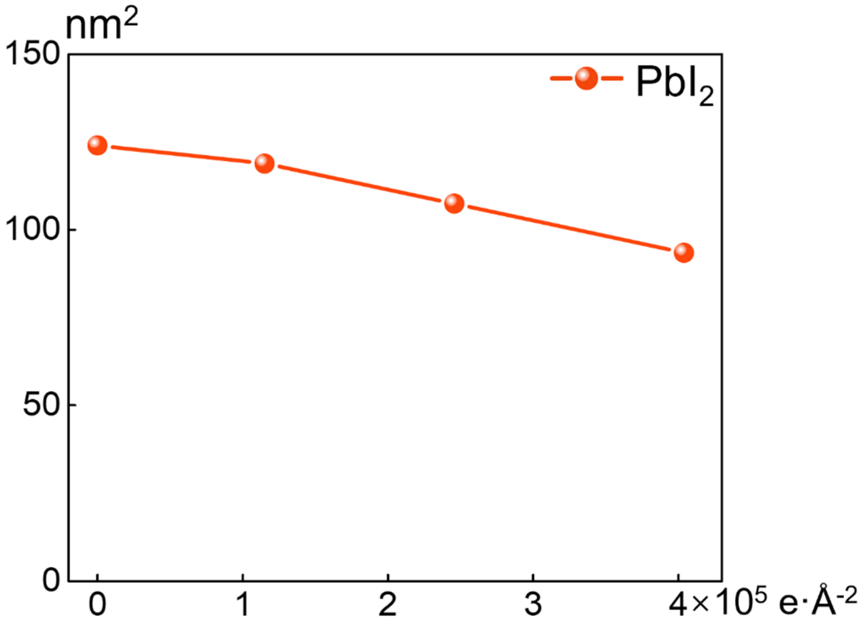

Supplementary Fig. 14 The size shrinkage of the PbI_2 impurity nanocluster with respect to the accumulated electron dose in Supplementary Fig. 12.

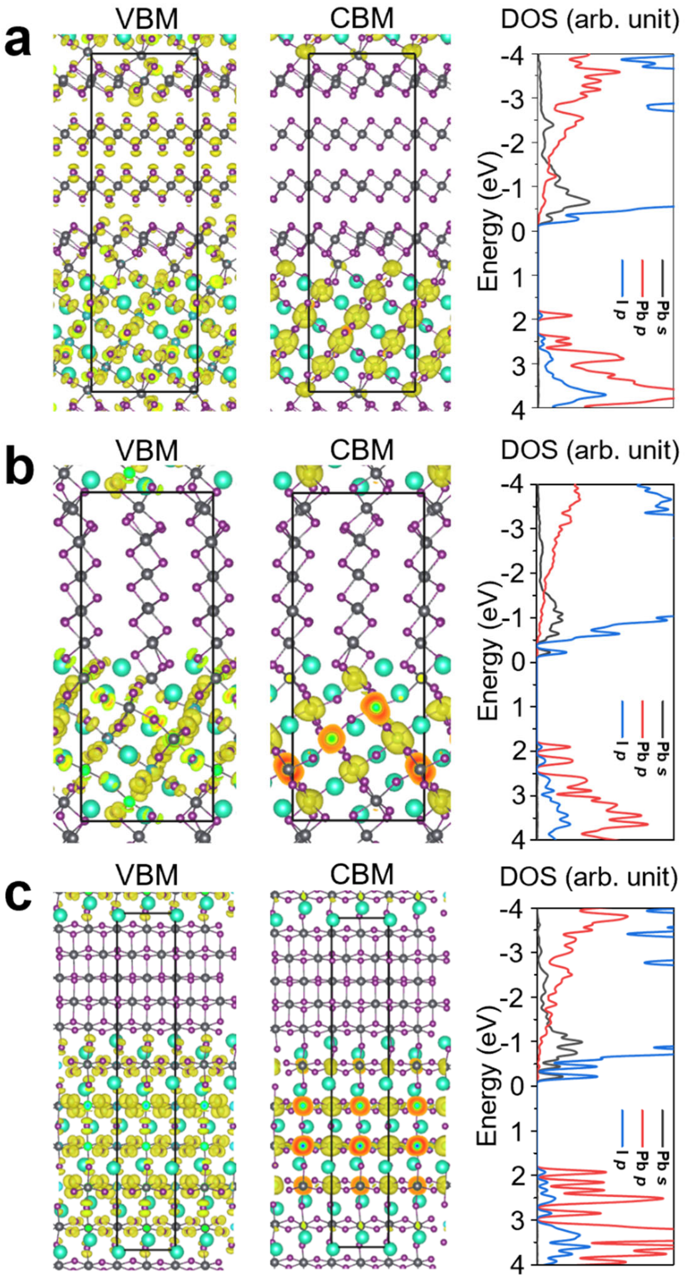

Supplementary Fig. 15 Partial DOS of the three intragrain PbI_2-perovskite interfaces. a-c, Charge density (yellow area) of the shoulder peak near the band edges and partial DOS for Pb *s, p* orbitals and I *p* orbitals for PbI_2-perovskite interfaces on the perovskite (01$\bar{1}$), (011) and (001) plane, respectively.

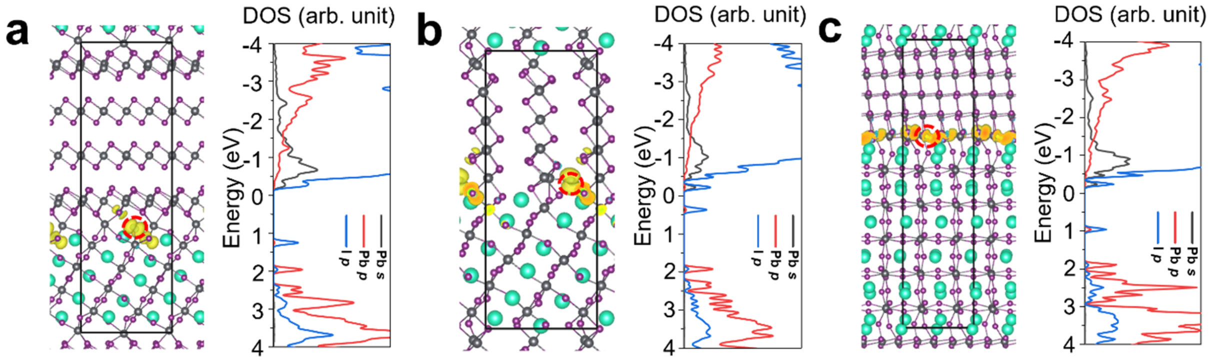

Supplementary Fig. 16 Partial DOS of the three intragrain PbI_2-perovskite interfaces with I interstitial. a-c, Charge density (yellow area) of the shoulder peak near the band edges and partial DOS for Pb *s, p* orbitals and I *p* orbitals for PbI_2-perovskite interfaces corresponds to Fig. 3d-f with one I interstitial (per supercell), respectively.

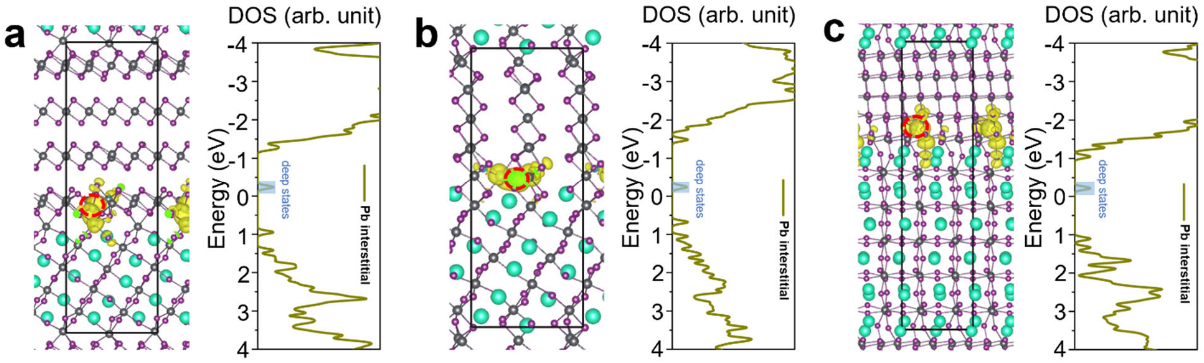

Supplementary Fig. 17 Electronic structures of the three intragrain PbI_2-perovskite interfaces with Pb interstitial. a-c, Charge density (yellow area) of the shoulder peak near the band edges and total DOS for PbI_2-perovskite interfaces along perovskite $(01\bar{1})$, (011) and (001) plane with one Pb interstitial (per supercell), respectively.

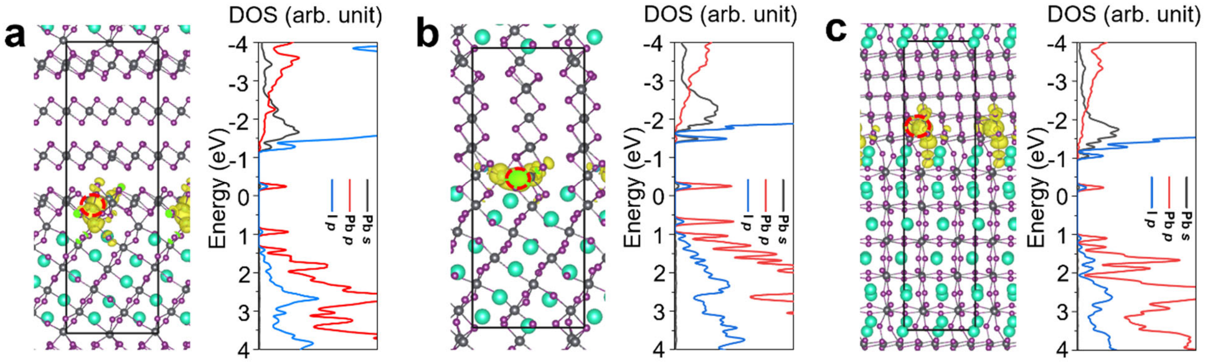

Supplementary Fig. 18 Partial DOS of the three intragrain PbI_2-perovskite interfaces with Pb interstitial. a-c, Charge density (yellow area) of the shoulder peak near the band edges and partial DOS for Pb *s, p* orbitals and I *p* orbitals for PbI_2-perovskite interfaces corresponds to Supplementary Fig. 16 with one Pb interstitial (per supercell), respectively.

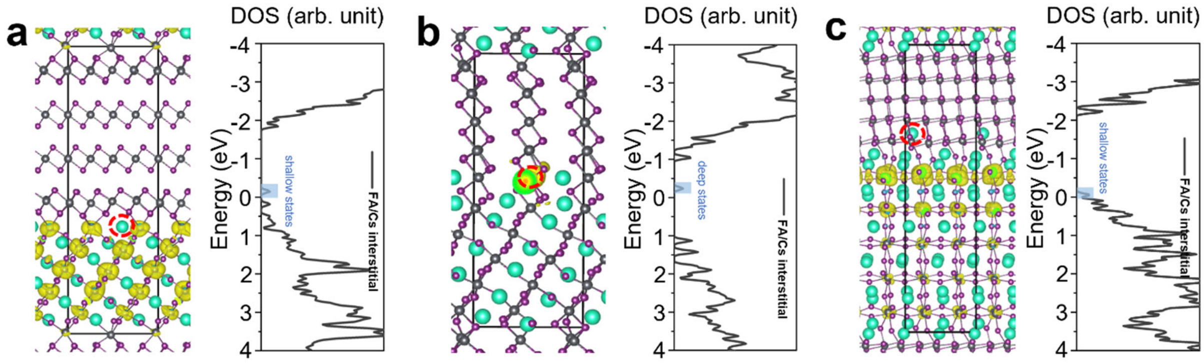

Supplementary Fig. 19 Electronic structures of the three intragrain PbI_2-perovskite interfaces with FA/Cs interstitial. a-c, Charge density (yellow area) of the shoulder peak near the band edges and total DOS for PbI_2-perovskite interfaces along perovskite (01$\bar{1}$), (011) and (001) plane with one FA/Cs interstitial (per supercell), respectively.

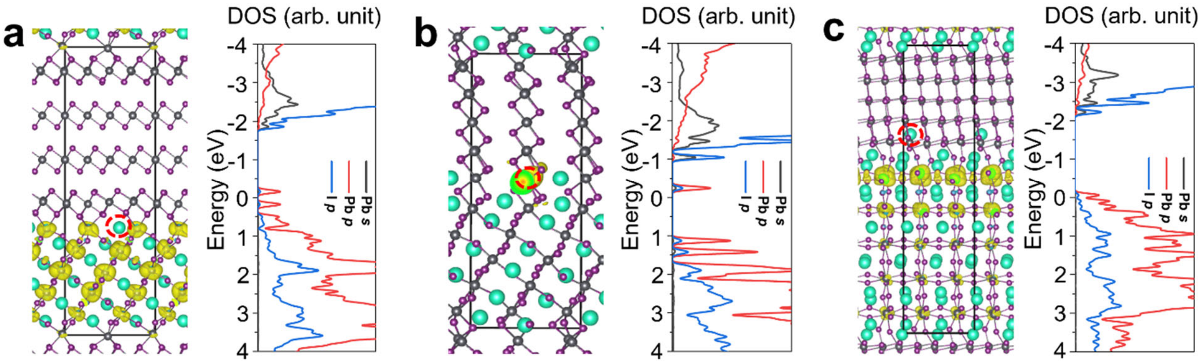

Supplementary Fig. 20 Partial DOS of the three intragrain PbI_2-perovskite interfaces with FA/Cs interstitial. a-c, Charge density (yellow area) of the shoulder peak near the band edges and partial DOS for Pb *s, p* orbitals and I *p* orbitals for PbI_2-perovskite interfaces corresponds to Supplementary Fig. 18 with one FA/Cs interstitial (per supercell), respectively.

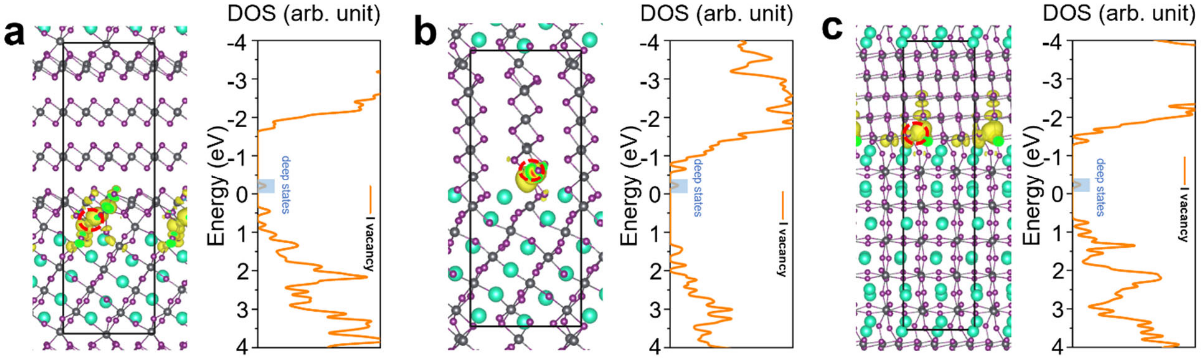

Supplementary Fig. 21 Electronic structures of the three intragrain PbI_2-perovskite interfaces with I vacancy. a-c, Charge density (yellow area) of the shoulder peak near the band edges and total DOS for PbI_2-perovskite interfaces along perovskite $(01\bar{1})$, (011) and (001) plane with one I vacancy (per supercell), respectively.

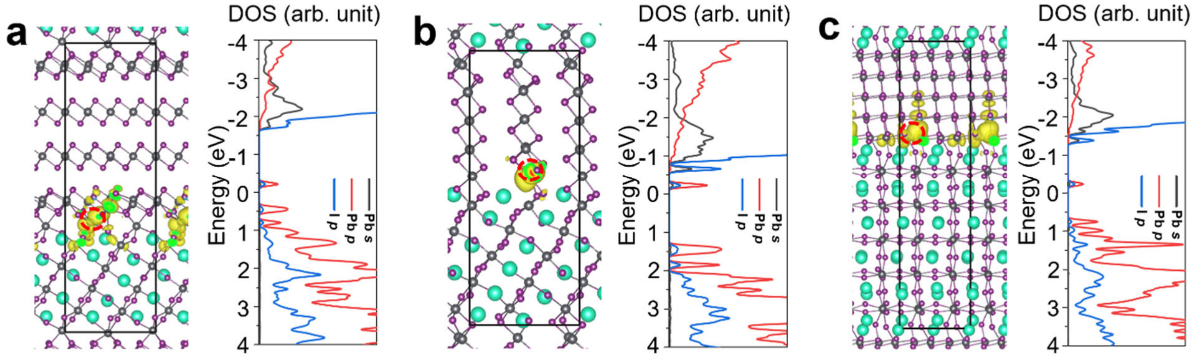

Supplementary Fig. 22 Partial DOS of the three intragrain PbI_2-perovskite interfaces with I vacancy. a-c, Charge density (yellow area) of the shoulder peak near the band edges and partial DOS for Pb *s, p* orbitals and I *p* orbitals for PbI_2-perovskite interfaces corresponds to Supplementary Fig. 20 with one I vacancy (per supercell), respectively.

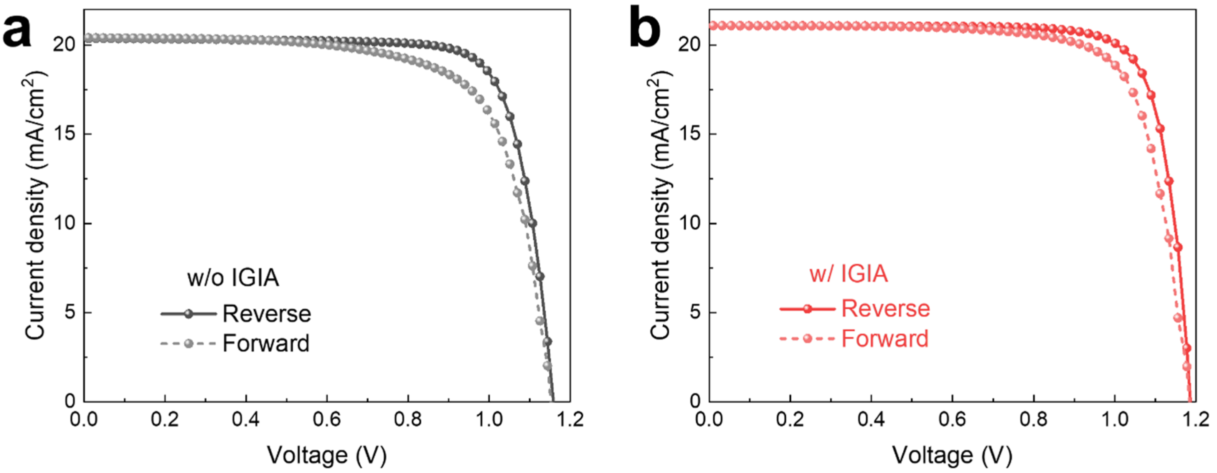

Supplementary Fig. 23 *J-V* hysteresis of an FA-Cs PSC before and after laser-induced IGIA. **a,** *J-V* hysteresis of an FA-Cs PSC before laser-induced IGIA. **b,** *J-V* hysteresis of FA-Cs PSC after laser-induced IGIA.

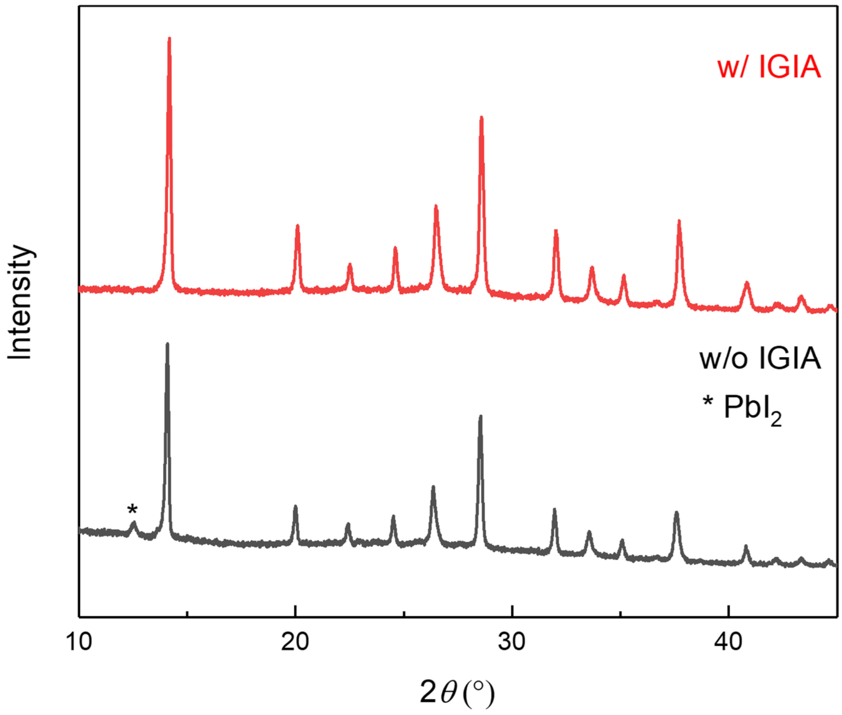

Supplementary Fig. 24 X-ray diffraction (XRD) patterns of $FA_{0.5}Cs_{0.5}PbI_3$ films before and after laser-induced IGIA.

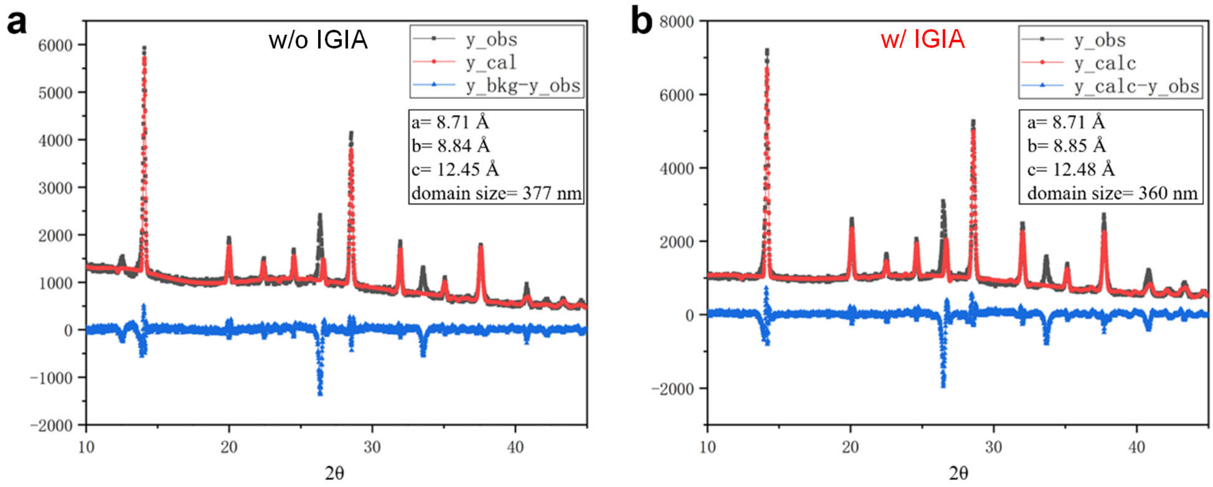

Supplementary Fig. 25 Refinement of XRD results presented in Supplementary Fig. 23. a, The lattice parameters of $FA_{0.5}Cs_{0.5}PbI_3$ perovskite film before laser-induced IGIA are measured as *a* = 8.71 Å, *b* = 8.84 Å, and *c* = 12.45 Å, and the crystalline domain size is estimated to be 377 nm from the XRD refinement. **b,** The lattice parameters of $FA_{0.5}Cs_{0.5}PbI_3$ perovskite film after laser treatment are measured as *a* = 8.71 Å, *b* = 8.85 Å, and *c* = 12.48 Å, and the crystalline domain size is estimated to be 360 nm from the XRD refinement. XRD refinements were carried out using Rietveld method with the GSAS (General Structure Analysis System) software [ref. [42,43]]. Note that the reliability factors (R_w) for refinements of XRD results obtained from film samples before and after laser-induced IGIA were 9.006 and 11.020, respectively.

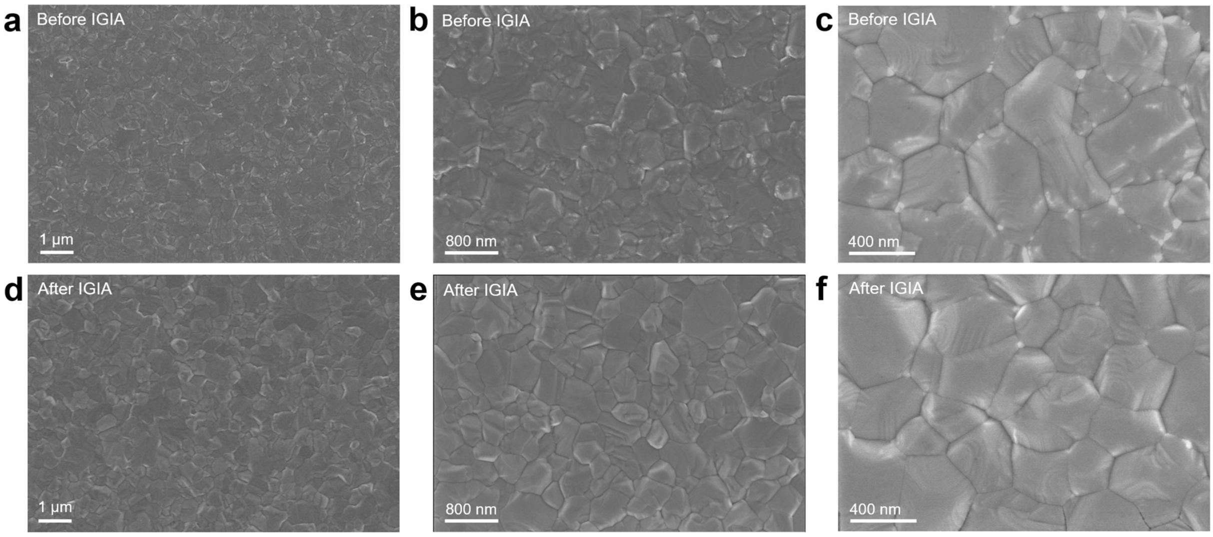

Supplementary Fig. 26 SEM images of the surface morphology of FA-Cs perovskite film before and after laser-induced IGIA. a-c, Low-magnification and high-magnification SEM images of the $FA_{0.5}Cs_{0.5}PbI_3$ perovskite film before laser-induced IGIA, respectively. **d-f,** Low-magnification and high-magnification SEM images of the $FA_{0.5}Cs_{0.5}PbI_3$ perovskite film after laser-induced IGIA, indicating little change in perovskite morphology.

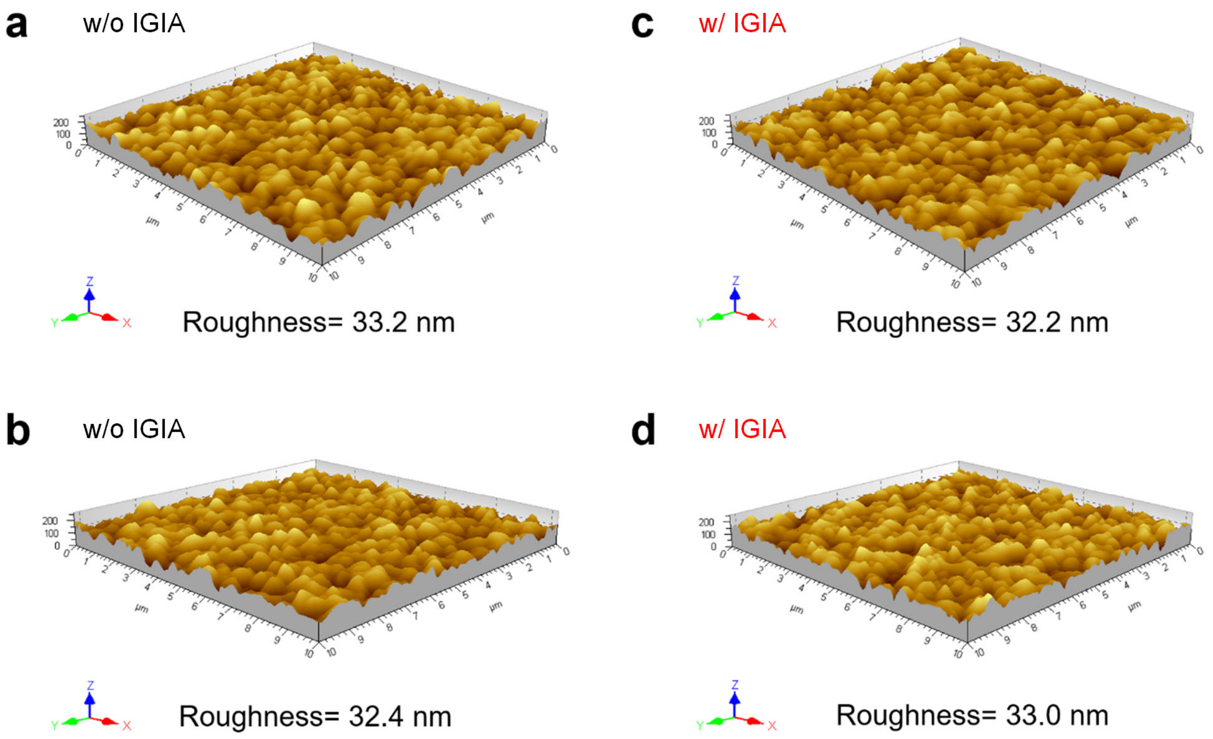

Supplementary Fig. 27 AFM measurement of the FA-Cs perovskite film surface roughness before and after laser-induced IGIA. a, b, AFM images of $FA_{0.5}Cs_{0.5}PbI_3$ perovskite film surface before laser-induced IGIA. **c, d,** AFM images of $FA_{0.5}Cs_{0.5}PbI_3$ perovskite film surface after laser-induced IGIA. Only slight variations in roughness are observed amongst these films.

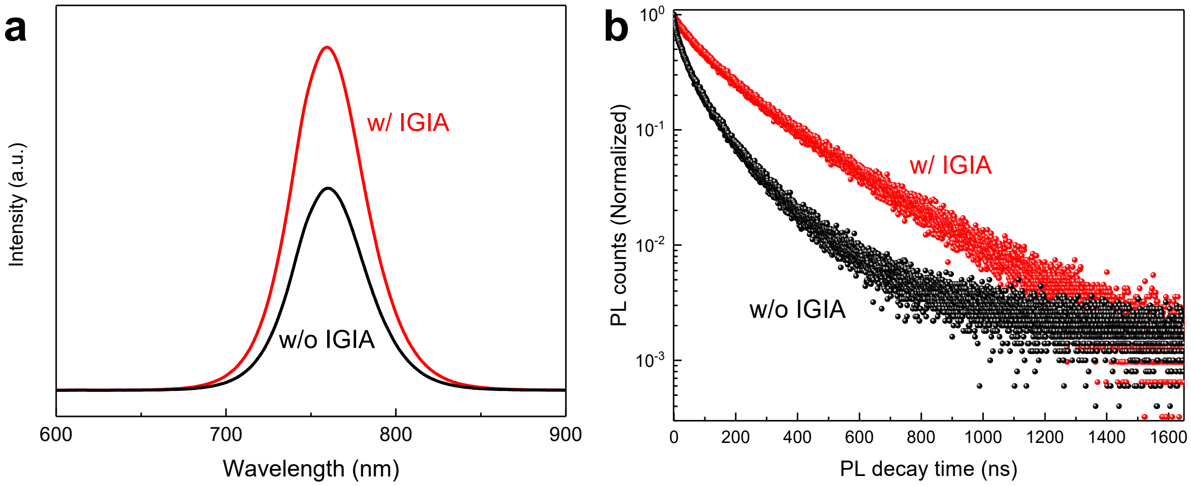

Supplementary Fig. 28 Steady-state photoluminescence (PL) and Time-resolved photoluminescence (TRPL) measurement of the FA-Cs perovskite film before and after laser-induced IGIA. a, PL spectra and **b**, TRPL spectra of $FA_{0.5}Cs_{0.5}PbI_3$ films before and after laser-induced IGIA, respectively.

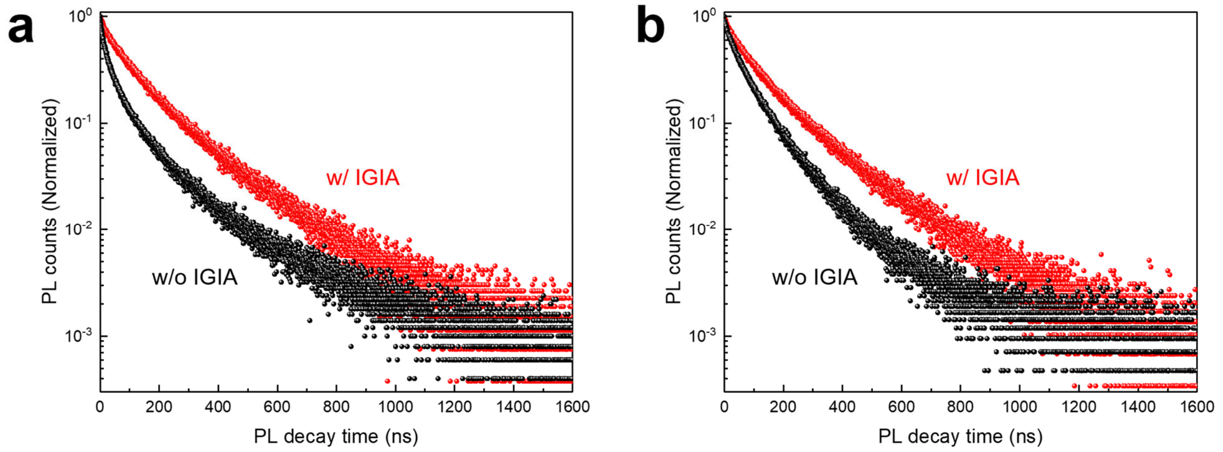

Supplementary Fig. 29 Time-resolved photoluminescence (TRPL) measurement of the FA-Cs perovskite film before and after laser-induced IGIA. a, b, TRPL spectra of $FA_{0.5}Cs_{0.5}PbI_3$ films before and after laser-induced IGIA measured from two different sites spaced at a distance of more than 5 mm.

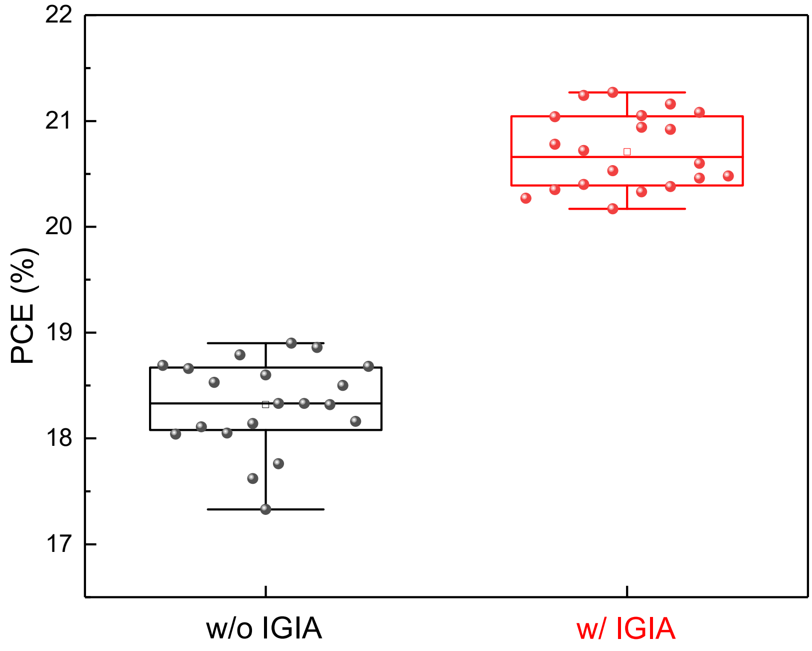

Supplementary Fig. 30 PCE statistics of 20 individual devices before and after laser-induced IGIA.

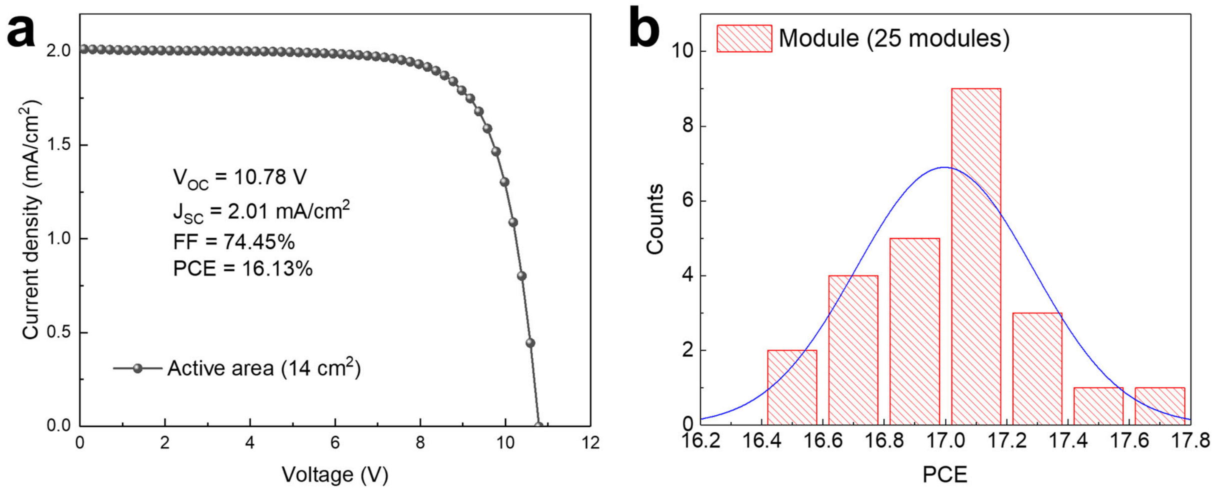

Supplementary Fig. 31 Current-voltage (*J-V*) curves and PCE of modules. a, *J-V* curve of a 14 cm^2 solar module before laser-induced IGIA. **b,** The PCE statistic of 25 modules after laser-induced IGIA.

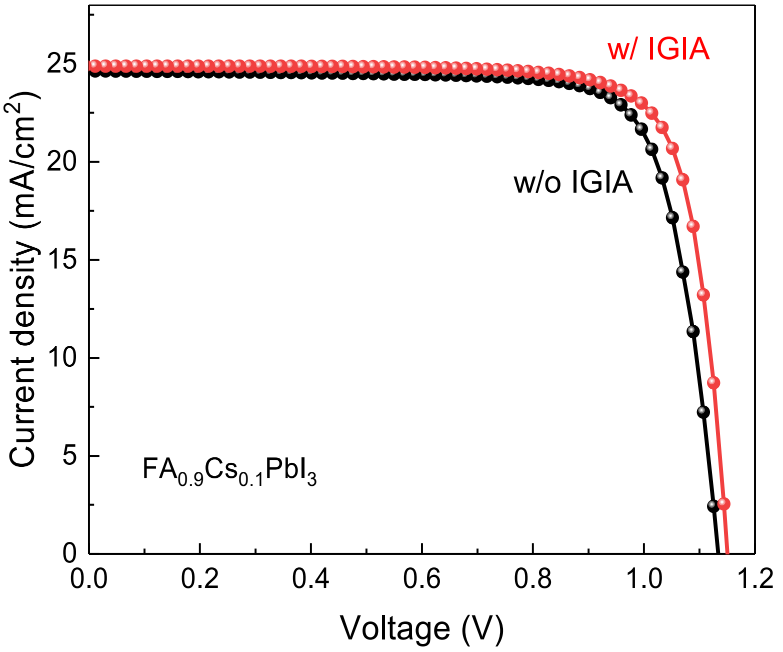

Supplementary Fig. 32 Current-voltage (*J-V*) curves of $FA_{0.9}Cs_{0.1}PbI_3$ PSCs before and after laser-induced intragrain phase-healing. The champion solar cell based on the target film displays a PCE of 22.87%, with open-circuit voltage (V_{OC}) of 1.15 V, short-circuit current density (J_{SC}) of 24.88 mA/cm^2, fill factor (FF) of 79.94%, demonstrating an obvious improvement, as compared with the 21.93% PCE of the control device (V_{OC}: 1.13 V; J_{SC}: 24.63 mA/cm^2; FF: 78.54%).

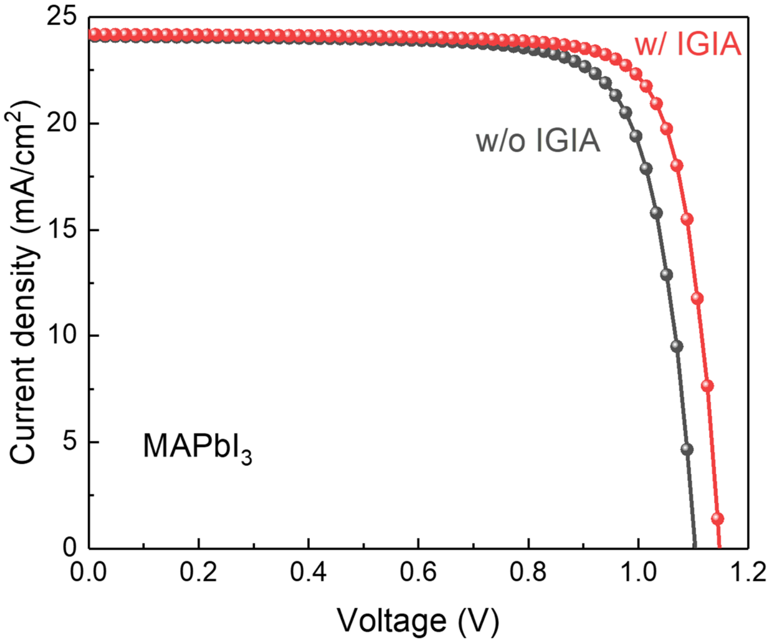

Supplementary Fig. 33 Current-voltage (*J-V*) curves of $MAPbI_3$ PSCs before and after laser-induced IGIA.

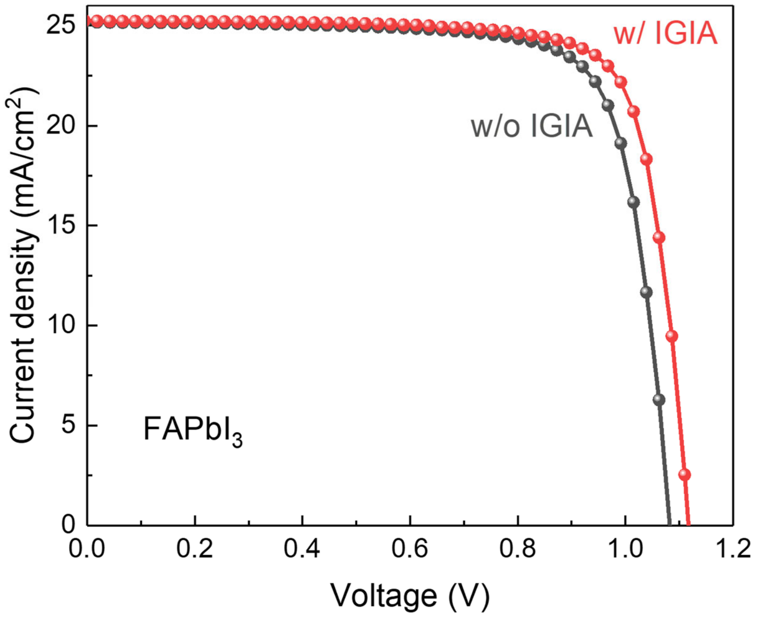

Supplementary Fig. 34 Current-voltage (*J*-*V*) curves of $FAPbI_3$ PSCs before and after laser-induced IGIA.

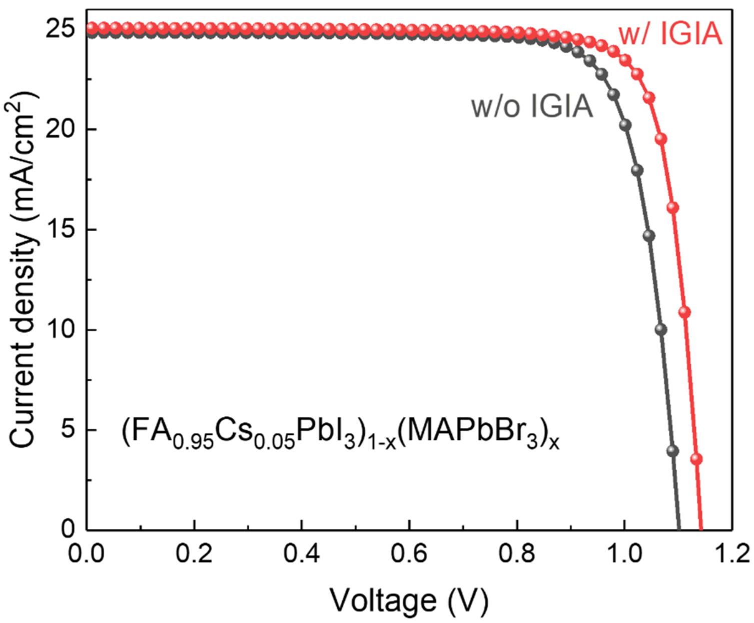

Supplementary Fig. 35 Current-voltage (*J*-*V*) curves of $(FA_{0.95}Cs_{0.05}PbI_3)_{1-x}(MAPbBr_3)_x$ PSCs before and after laser-induced IGIA .

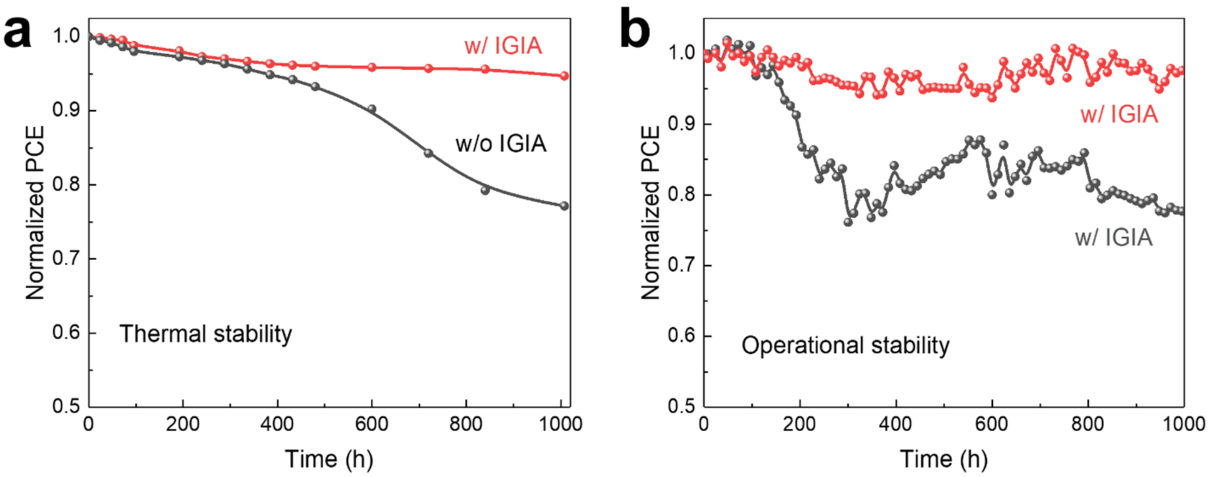

Supplementary Fig. 36 Thermal stability and operational stability tests of FA-Cs PSCs before and after laser-induced IGIA. a, Thermal stability of FA-Cs PSC with and without laser-induced IGIA tested at 85 °C for 1000 h. **b,** Operational stability of FA-Cs PSC with and without laser-induced IGIA tested under one-sun illumination for 1000 h.

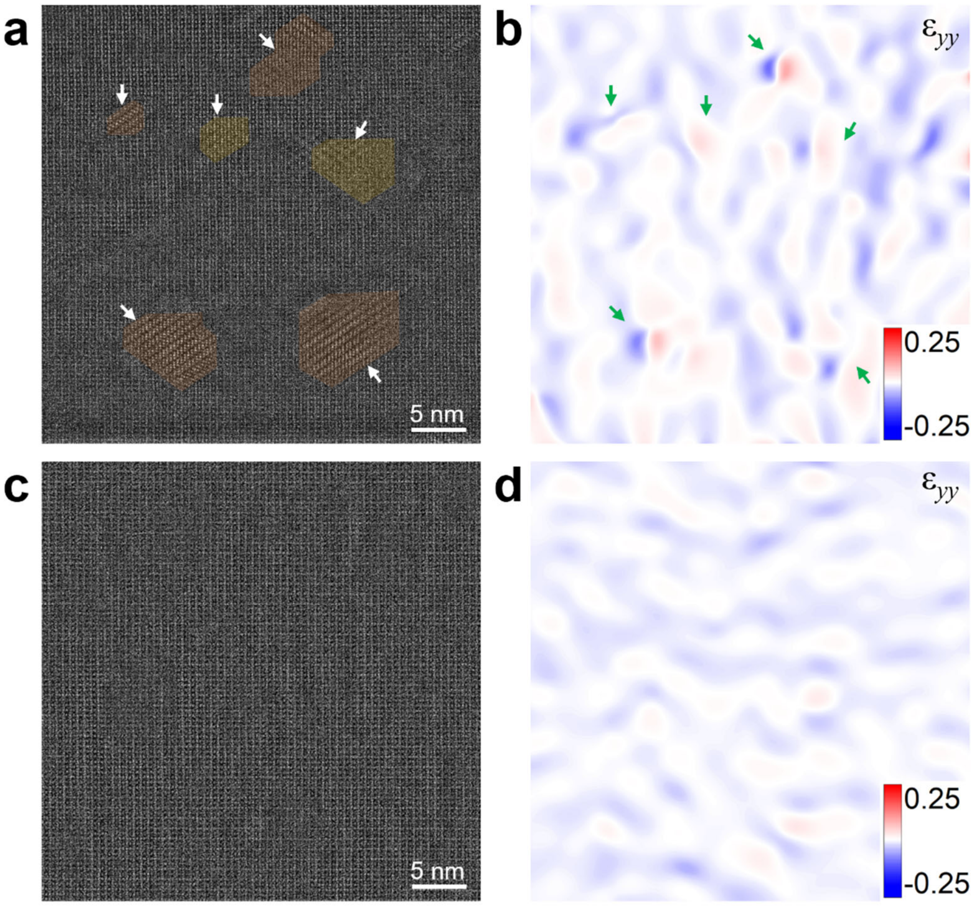

Supplementary Fig. 37 Perovskite intragrain strain relaxation coupled with laser-induced IGIA. a, Atomic-resolution STEM-HAADF image of pristine perovskite grain in as-fabricated PSC sample contains both PbI_2 and non-PbI_2 impurity nanoclusters. **b,** GPA mapping of out-of-plane strain (ε_{yy}) distribution in **a**, indicating the PbI_2 nanocluster and non-PbI_2 impurity phase induced uneven intragrain strain as marked by green arrows. **c,** STEM-HAADF image of PSC sample after laser-induced IGIA with a reduced impurity density. **d,** GPA mapping of out-of-plane strain (ε_{yy}) distribution in **c**, showing that intragrain strain is effectively relaxed.

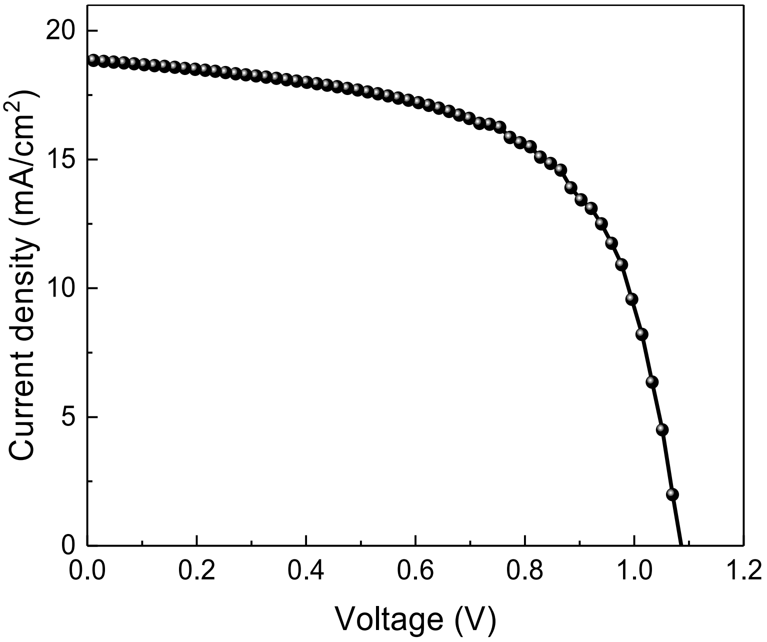

Supplementary Fig. 38 Current-voltage (*J*-*V*) curves of $FA_{0.5}Cs_{0.5}PbI_3$ PSC after laser treatment for 10 min (PCE: 12.5%; V_{OC}: 1.08 V; J_{SC}: 18.85 mA/cm^2; FF: 61.40%).

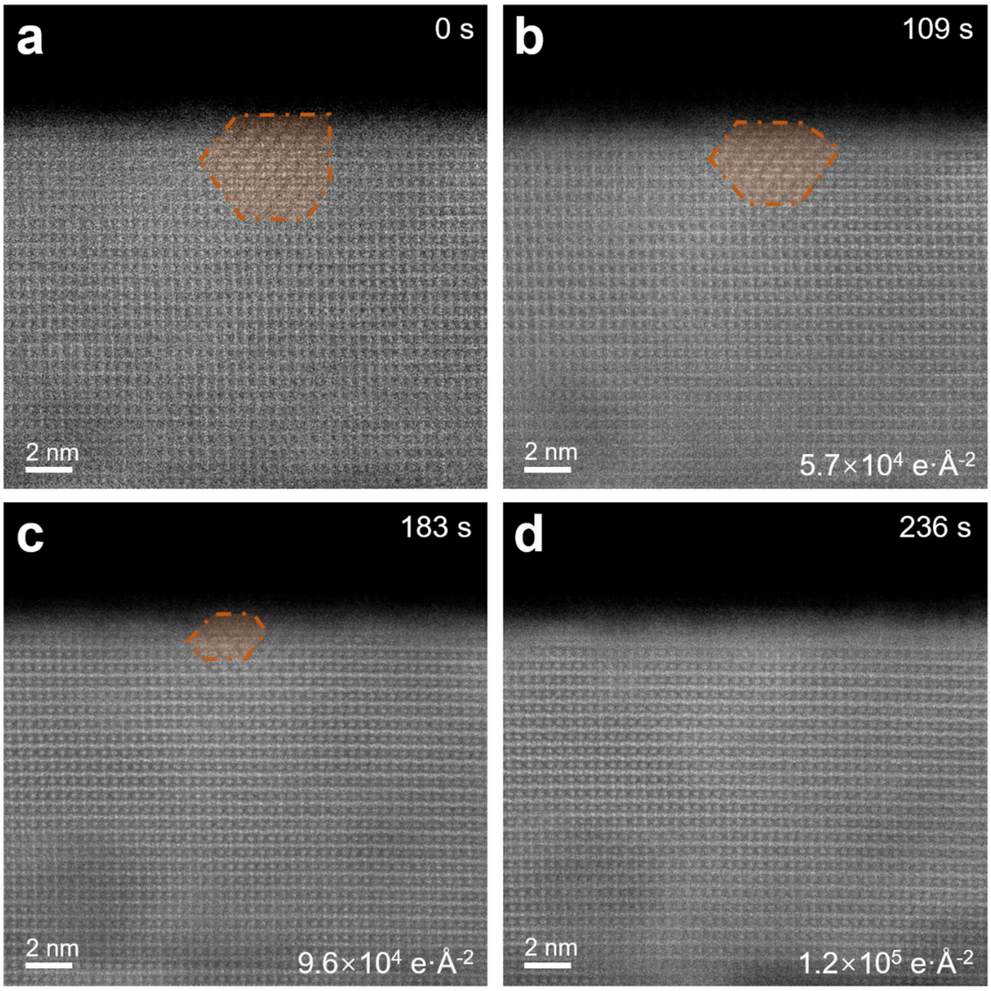

Supplementary Fig. 39 *In situ* STEM observation of intragrain impurity annihilation in MA-Cs perovskite. a, A PbI_2 nanocluster locates near the surface of $MA_{0.5}Cs_{0.5}PbI_3$ perovskite grain. **b-d,** The shrinkage and annihilation of this PbI_2 nanocluster under electron probe scanning.

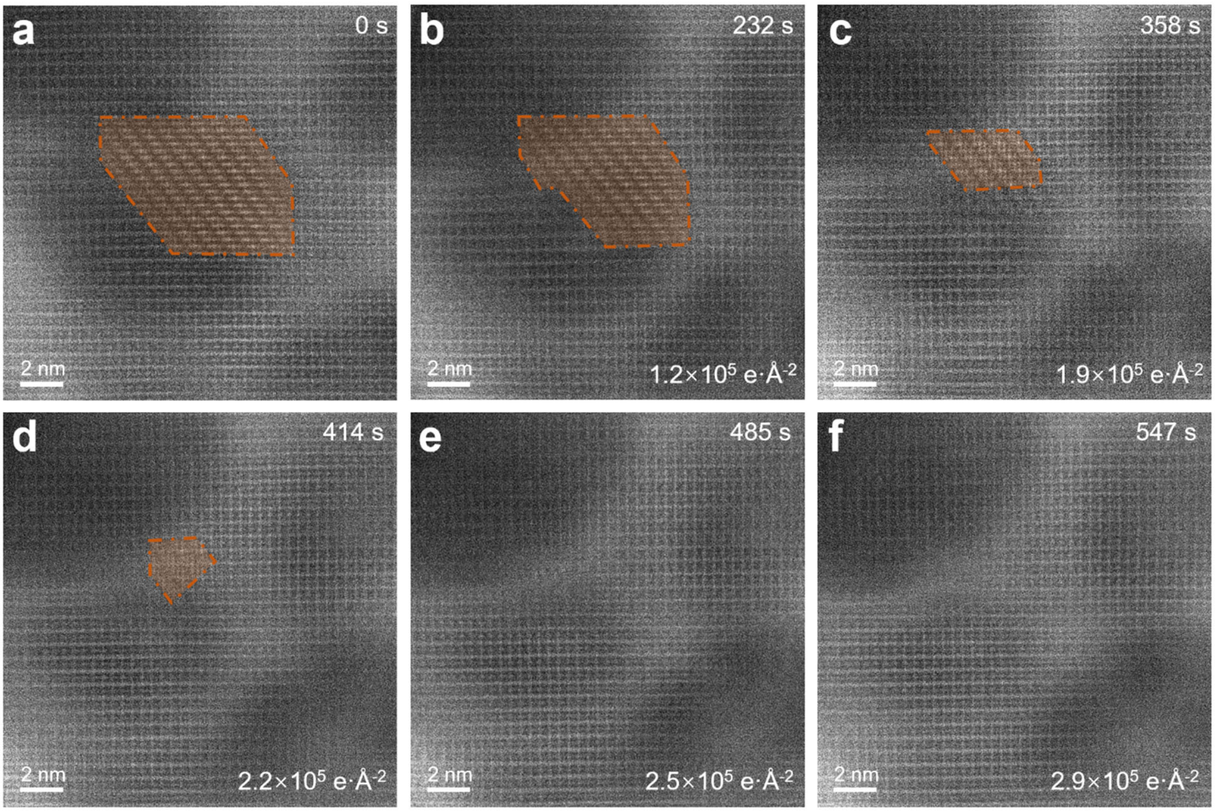

Supplementary Fig. 40 *In situ* STEM observation of intragrain impurity annihilation in MA-Cs perovskite. a, A PbI_2 nanocluster locates in the inner region of $MA_{0.5}Cs_{0.5}PbI_3$ perovskite grain. **b-f,** The shrinkage and annihilation of this PbI_2 nanocluster under electron probe scanning.

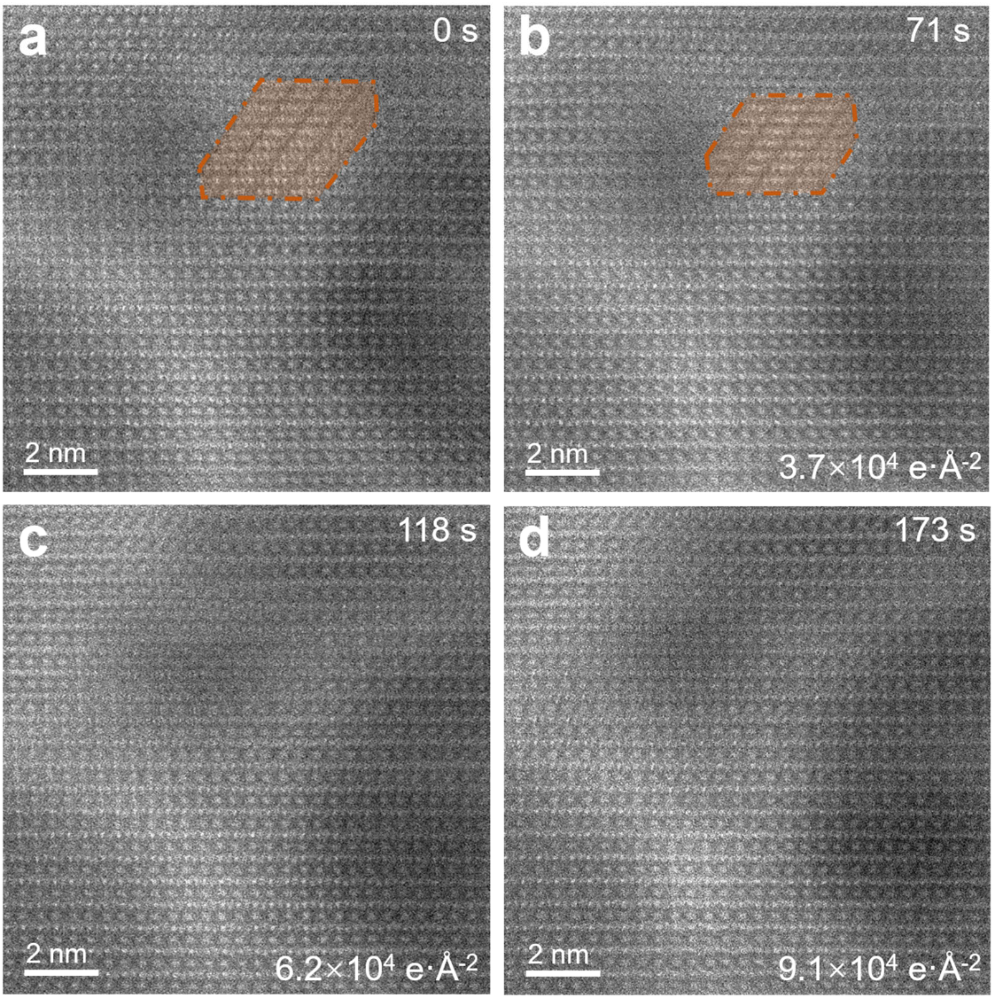

Supplementary Fig. 41 *In situ* STEM observation of intragrain impurity annihilation in over-annealed FA-Cs perovskite. a, A PbI_2 nanocluster exists in $FA_{0.5}Cs_{0.5}PbI_3$ perovskite grain prepared by 140 °C, 90 min annealing. **b-d,** The shrinkage and annihilation of this PbI_2 nanocluster under electron probe scanning.

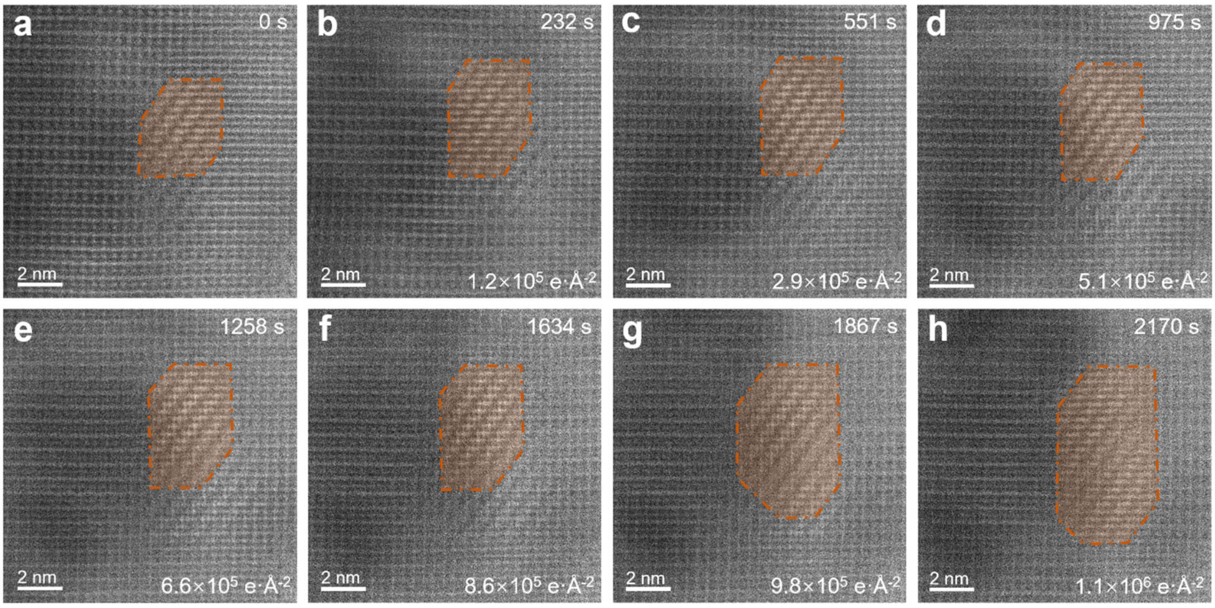

Supplementary Fig. 42 *In situ* STEM observation of intragrain impurity evolution in FA-Cs perovskite prepared without FACl incorporation. a, A PbI_2 nanocluster exists in $FA_{0.5}Cs_{0.5}PbI_3$ perovskite grain prepared without FACl incorporation. **b-h,** The slight expansion of this PbI_2 nanocluster under electron probe scanning.

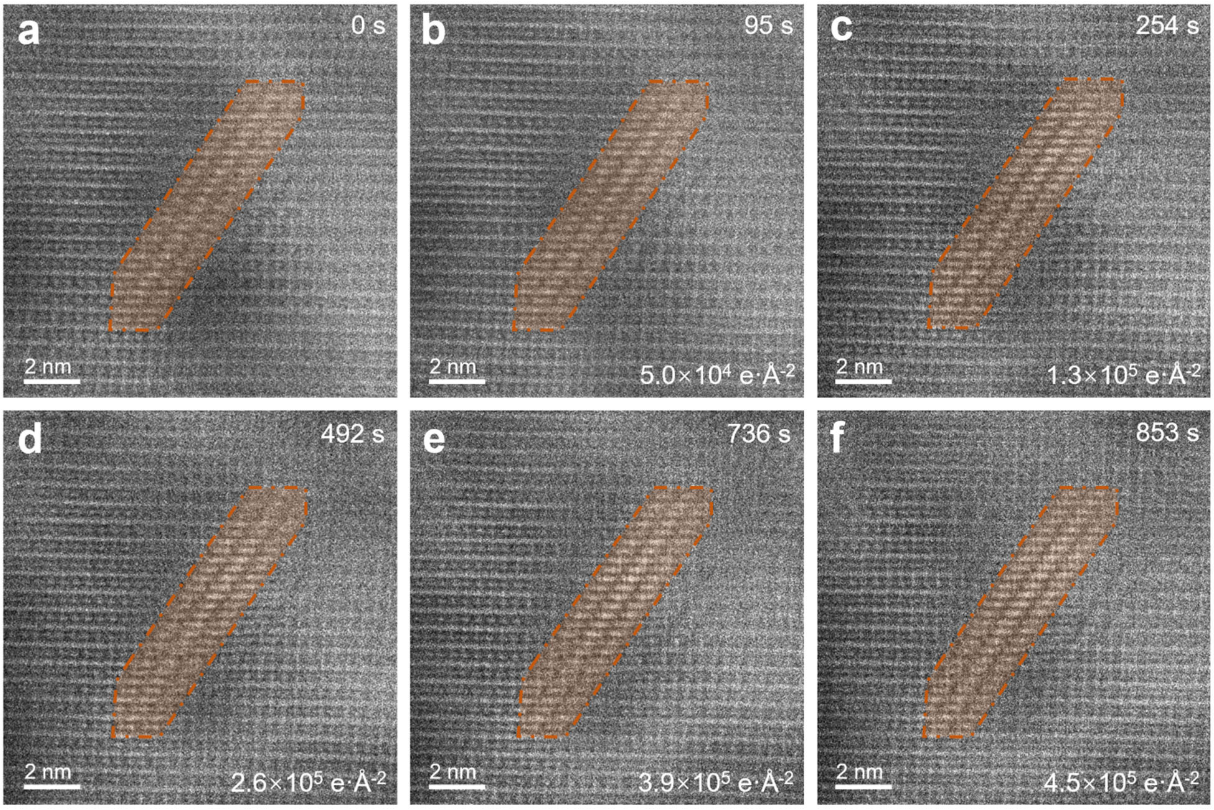

Supplementary Fig. 43 *In situ* STEM observation of intragrain impurity evolution in MA-Cs perovskite prepared without MACl incorporation. a, A PbI_2 nanocluster exists in $MA_{0.5}Cs_{0.5}PbI_3$ perovskite grain prepared without MACl incorporation. **b-f,** The size of this PbI_2 nanocluster exhibits little change under electron probe scanning.

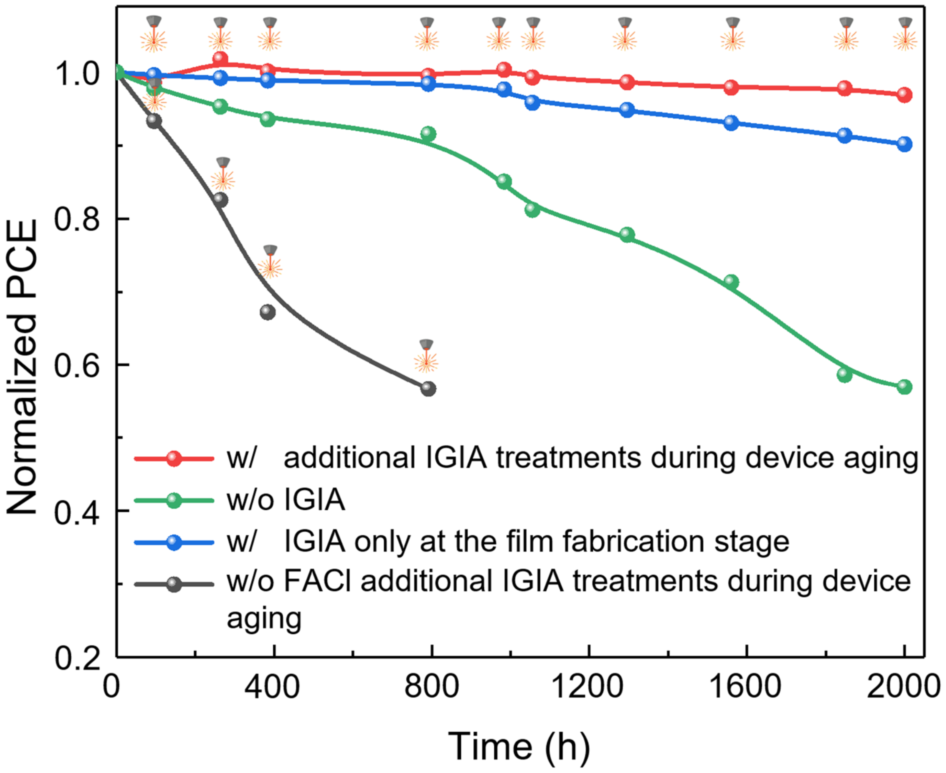

Supplementary Fig. 44 Stability test of FA-Cs PSCs with different laser treatment and synthesis conditions. The red, blue and green curves are same with Fig. 4f. The black curve represents the result of PSC device prepared from $FA_{0.5}Cs_{0.5}PbI_3$ perovskite prepared without FACl incorporation, and the laser treatment has no effect compared with the normally prepared devices (red and blue curves).

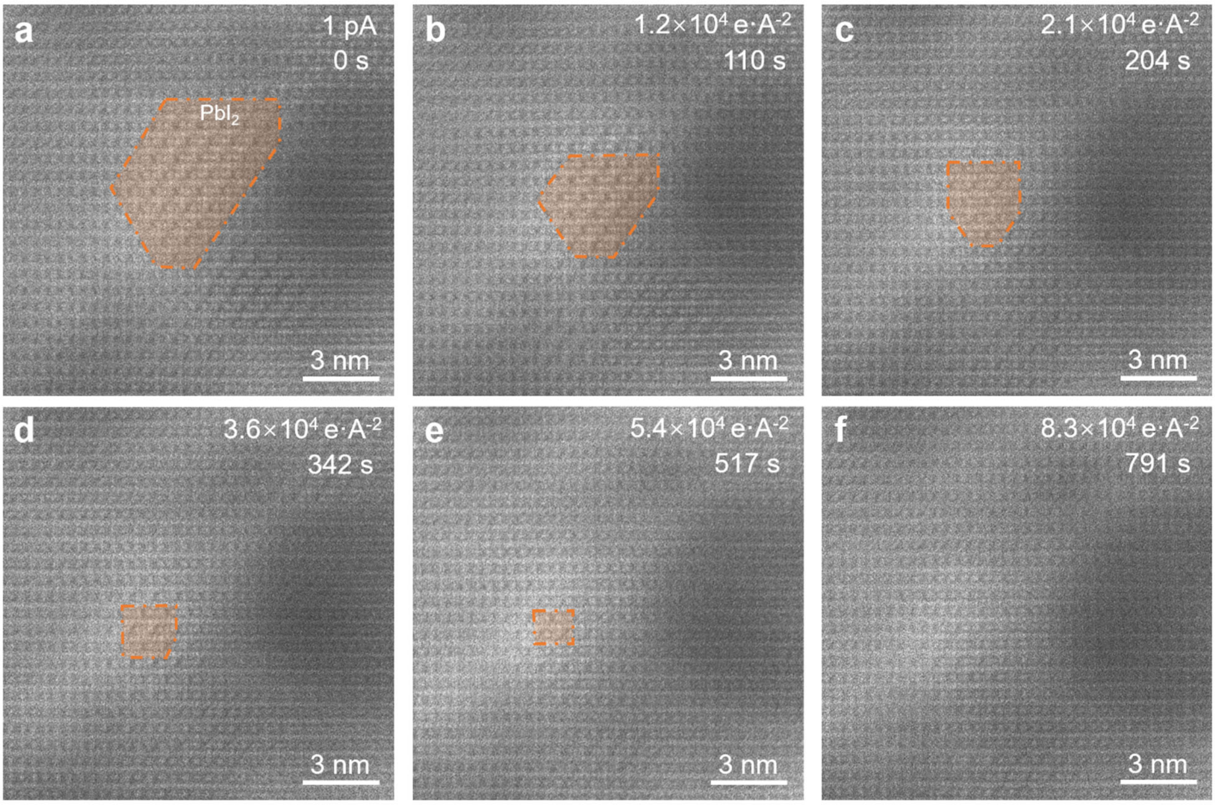

Supplementary Fig. 45 *In situ* STEM observation of intragrain impurity annihilation under smaller electron beam current. a, A PbI_2 nanocluster exists in $FA_{0.5}Cs_{0.5}PbI_3$ perovskite grain. **b-f,** The shrinkage and annihilation of this PbI_2 nanocluster under 1 pA electron probe scanning.

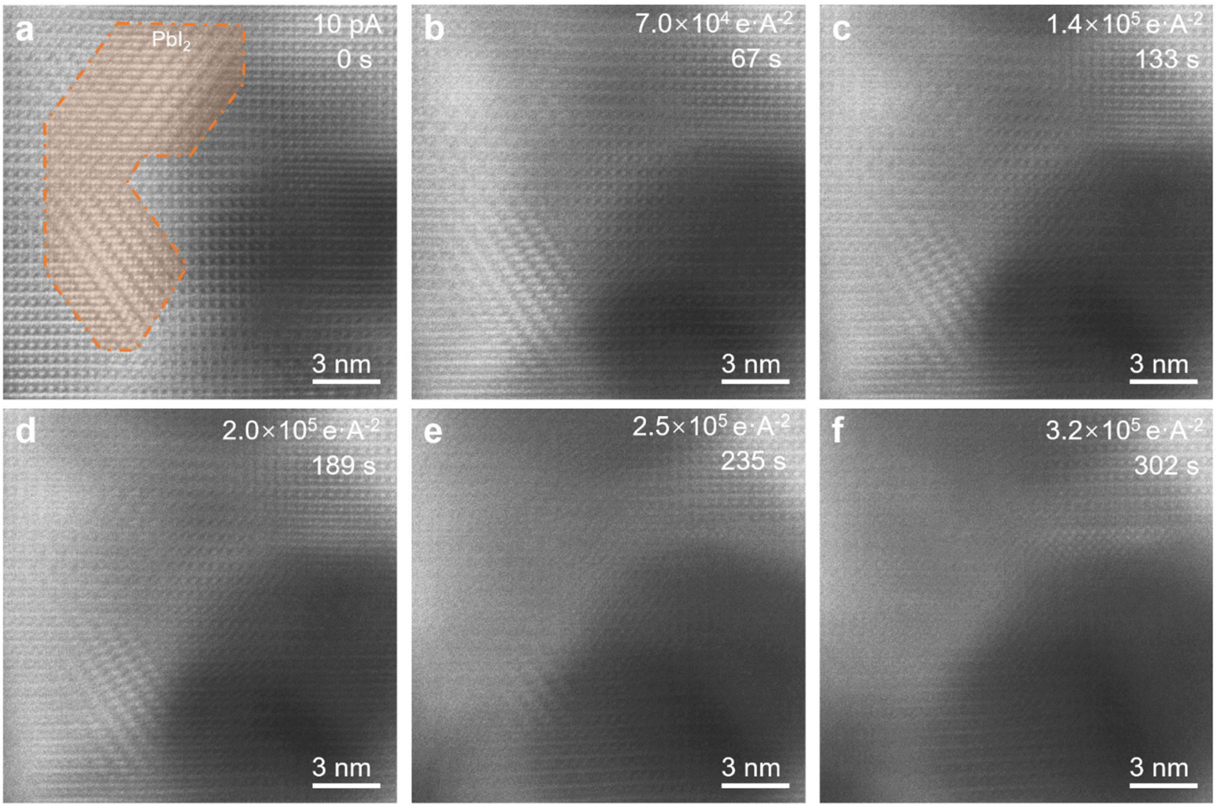

Supplementary Fig. 46 *In situ* STEM observation of intragrain impurity evolution under larger electron beam current. a, A PbI_2 nanocluster exists in $FA_{0.5}Cs_{0.5}PbI_3$ perovskite grain. **b-f,** The perovskite structure gradually collapse under 10 pA electron probe scanning.

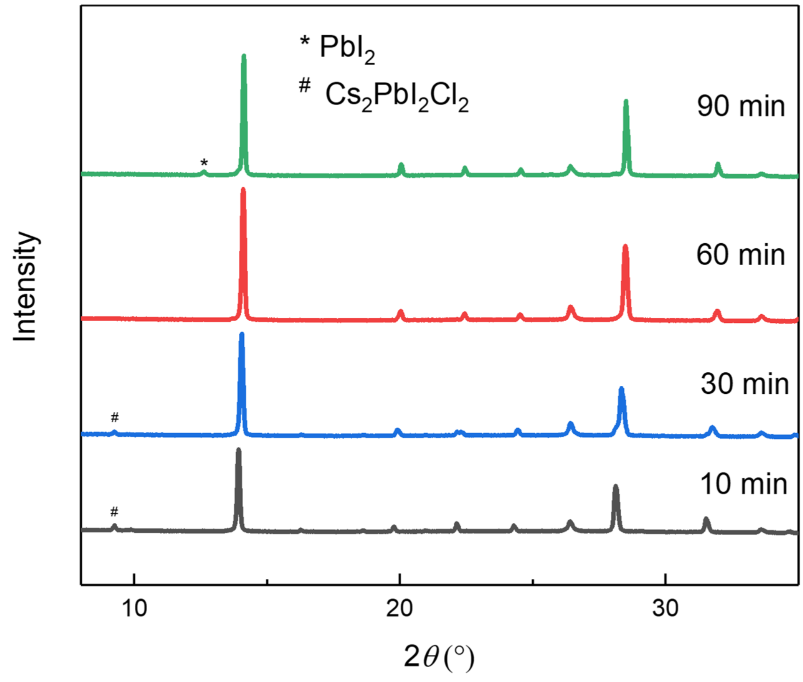

Supplementary Fig. 47 XRD patterns of $FA_{0.5}Cs_{0.5}PbI_3$ films prepared with different annealing times.

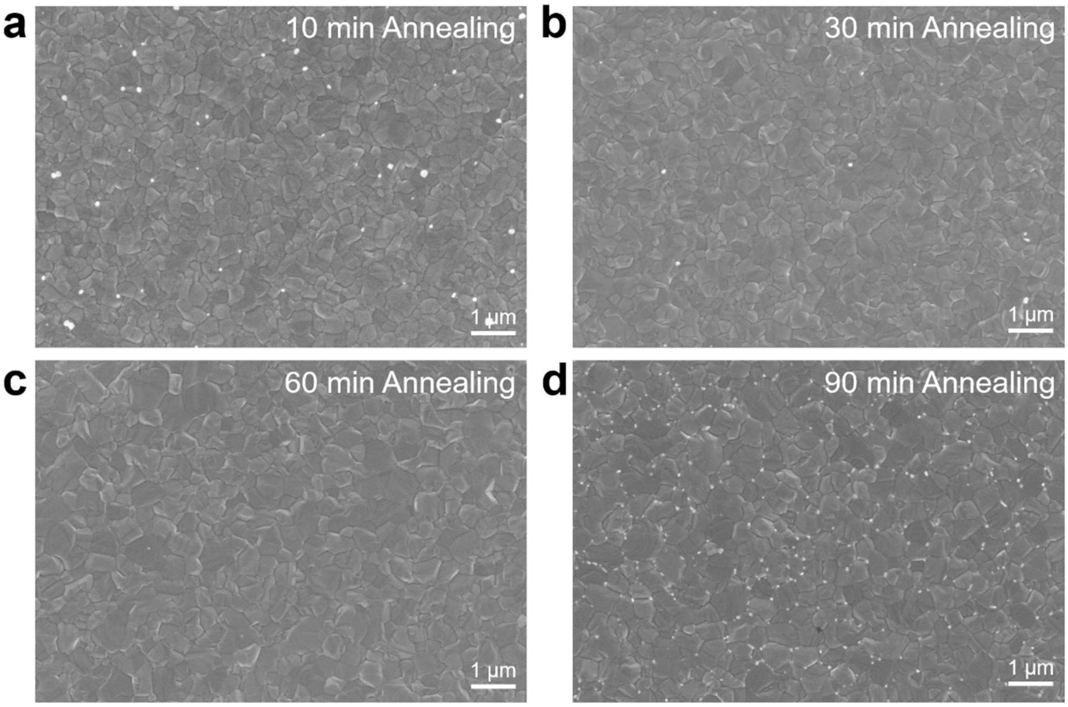

Supplementary Fig. 48 Top-view SEM images of $FA_{0.5}Cs_{0.5}PbI_3$ perovskite films with different annealing times. a-d, Top-view SEM images of perovskite films annealed for 10 min, 30 min, 60 min and 90 min, respectively. Some large bright-contrast clusters exist in the 10 min (**a**) and 30 min (**b**) cases. For the 90 min case (**d**), numerous bright-contrast dots appear onto the film surface.

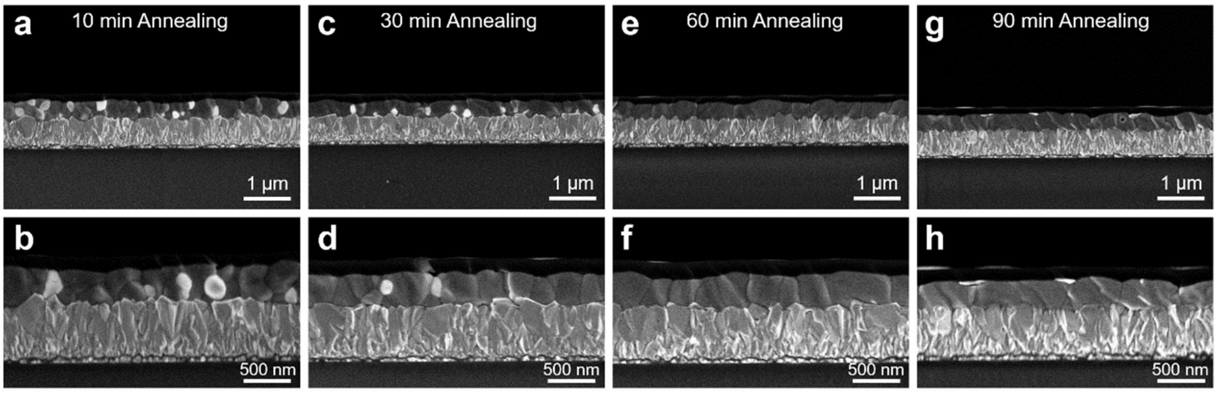

Supplementary Fig. 49 Cross-sectional SEM images of $FA_{0.5}Cs_{0.5}PbI_3$ perovskite films made with different annealing times. a-h, Cross-sectional SEM images of perovskite films that are annealed for 10 min (**a**, **b**), 30 min (**c**, **d**), 60 min (**e**, **f**) and 90 min (**g**, **h**), respectively. For the 10 min (**a, b**) and 30 min (**c, d**) cases, some large bright-contrast clusters exist in the film bulk. For the 90 min case (**g, h**), only some bright-contrast dots appear onto the film surface.

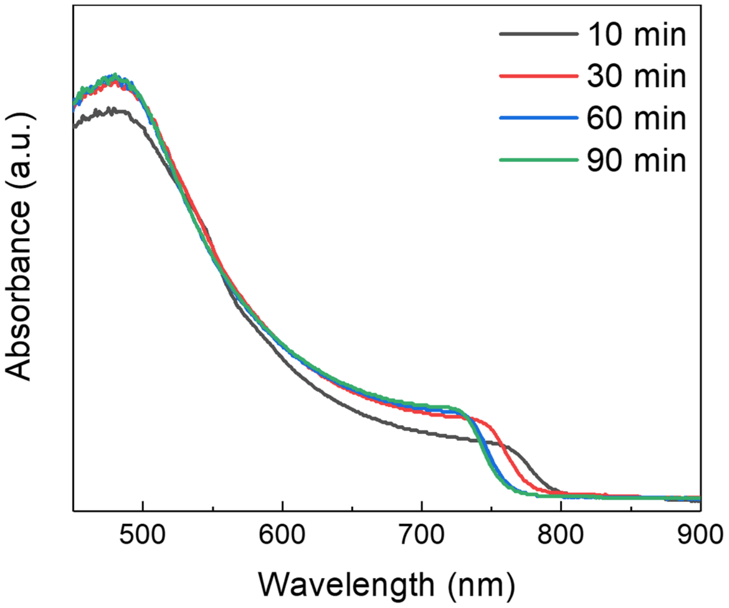

Supplementary Fig. 50 UV-*vis* spectra of $FA_{0.5}Cs_{0.5}PbI_3$ perovskite films made with different annealing times.

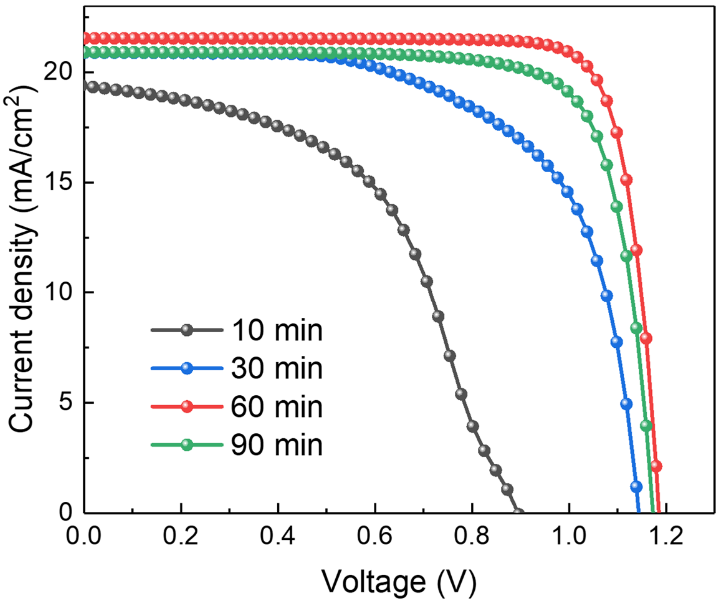

Supplementary Fig. 51 Current-voltage (*J*-*V*) curves of PSCs fabricated using $FA_{0.5}Cs_{0.5}PbI_3$ perovskite films made with different annealing times.

Supplementary Table 1. The calculated carrier lifetime values.

	**τ_1（ns）**	**τ_2（ns）**
w/o IGIA	23.1	114.1
w/ IGIA	63.8	224.3

Supplementary Table 2. The calculated carrier lifetime values.

System	w/o	V_{oc}	J_{sc}	FF	PCE
$MAPbI_3$	w/o	1.10	24.09	77.52	20.60
	w/	1.15	24.18	80.09	22.20
$FAPbI_3$	w/o	1.08	25.16	77.61	21.11
	w/	1.12	25.22	79.10	22.34
(FA,Cs)Br	w/o	1.10	24.84	80.11	21.90
	w/	1.14	25.06	82.01	23.43
