# Supplementary material for: Intragrain impurity annihilation for highly efficient and stable perovskite solar cells
Source: Nat Commun. 2024 Mar 14;15:2329. doi: 10.1038/s41467-024-46588-y (PMC10940583; doi:10.1038/s41467-024-46588-y)
Supplement: Supplementary file 2 — Reporting Summary [file 41467_2024_46588_MOESM2_ESM.pdf]

Corresponding author(s): Songhua Cai, Ming-Gang Ju, Shuping Pang, Yuanyuan Zhou

Solar Cells Reporting Summary

Nature Research wishes to improve the reproducibility of the work that we publish. This form is intended for publication with all accepted papers reporting the characterization of photovoltaic devices and provides structure for consistency and transparency in reporting. Some list items might not apply to an individual manuscript, but all fields must be completed for clarity.

For further information on Nature Research policies, including our data availability policy, see Authors & Referees.

Experimental design

Please check: are the following details reported in the manuscript?

1. Dimensions

Item	Yes/No	Details
Area of the tested solar cells	☒ Yes ☐ No	Provided in the Methods
Method used to determine the device area	☒ Yes ☐ No	Provided in the Methods

2. Current-voltage characterization

Item	Yes/No	Details
Current density-voltage (J-V) plots in both forward and backward direction	☒ Yes ☐ No	Provided in the Supplementary Information (Supplementary Figure 22)
Voltage scan conditions<br>*For instance: scan direction, speed, dwell times*	☒ Yes ☐ No	Provided in the Methods
Test environment<br>*For instance: characterization temperature, in air or in glove box*	☒ Yes ☐ No	Provided in the Methods
Protocol for preconditioning of the device before its characterization	☐ Yes ☒ No	There is no protocol for preconditioning of the device before its characterization
Stability of the J-V characteristic<br>*Verified with time evolution of the maximum power point or with the photocurrent at maximum power point; see ref. 7 for details.*	☒ Yes ☐ No	Provided in the Methods

3. Hysteresis or any other unusual behaviour

Item	Yes/No	Details
Description of the unusual behaviour observed during the characterization	☒ Yes ☐ No	Described in the manuscript
Related experimental data	☒ Yes ☐ No	See Supplementary Figure 23

4. Efficiency

Item	Yes/No	Details
External quantum efficiency (EQE) or incident photons to current efficiency (IPCE)	☒ Yes ☐ No	See Figure 4d.
A comparison between the integrated response under the standard reference spectrum and the response measure under the simulator	☒ Yes ☐ No	Provided in the Methods
For tandem solar cells, the bias illumination and bias voltage used for each subcell	☐ Yes ☒ No	There is no tandem solar cells.

5. Calibration

Item	Yes/No	Details
Light source and reference cell or sensor used for the characterization	☒ Yes ☐ No	Provided in the Methods
Confirmation that the reference cell was calibrated and certified	☒ Yes ☐ No	Provided in the Methods

Calculation of spectral mismatch between the reference cell and the devices under test

☒ Yes ☐ No — Provided in the Methods

6. Mask/aperture

Size of the mask/aperture used during testing

☒ Yes ☐ No — Provided in the Methods

Variation of the measured short-circuit current density with the mask/aperture area

☐ Yes ☒ No — We have not compare the short-circuit current density with or without aperture area.

7. Performance certification

Identity of the independent certification laboratory that confirmed the photovoltaic performance

☐ Yes ☒ No — There is no performance certification data.

A copy of any certificate(s)
Provide in Supplementary Information

☐ Yes ☒ No — There is no performance certification data.

8. Statistics

Number of solar cells tested

☒ Yes ☐ No — See Supplementary Figure 28 and 29).

Statistical analysis of the device performance

☒ Yes ☐ No — Provided in Supplementary Information (Supplementary Figures 30 and 31).

9. Long-term stability analysis

Type of analysis, bias conditions and environmental conditions
For instance: illumination type, temperature, atmosphere humidity, encapsulation method, preconditioning temperature

☒ Yes ☐ No — See Figure 4f.
